# A single-input binary counting module based on serine integrase site-specific recombination

**DOI:** 10.1093/nar/gkz245

**Published:** 2019-04-08

**Authors:** Jia Zhao, Alexandra Pokhilko, Oliver Ebenhöh, Susan J Rosser, Sean D Colloms

**Affiliations:** 1 Institute of Molecular, Cell and Systems Biology, University of Glasgow, Bower Building, Glasgow G12 8QQ, Scotland; 2 Cluster of Excellence on Plant Sciences (CEPLAS), Heinrich-Heine-University, Universitätsstraße 1, D-40225 Düsseldorf, Germany; 3 Institute of Quantitative and Theoretical Biology, Heinrich-Heine-University Düsseldorf, Universitätsstraße 1, D-40225 Düsseldorf, Germany; 4 SynthSys - Synthetic and Systems Biology, School of Biological Sciences, University of Edinburgh, CH Waddington Building, The King’s Buildings, Mayfield Road, Edinburgh EH9 3JD, Scotland

## Abstract

A device that counts and records the number of events experienced by an individual cell could have many uses in experimental biology and biotechnology. Here, we report a DNA-based ‘latch’ that switches between two states upon each exposure to a repeated stimulus. The key component of the latch is a DNA segment whose orientation is inverted by the actions of ϕC31 integrase and its recombination directionality factor (RDF). Integrase expression is regulated by an external input, while RDF expression is controlled by the state of the latch, such that the orientation of the invertible segment switches efficiently each time the device receives an input pulse. Recombination occurs over a time scale of minutes after initiation of integrase expression. The latch requires a delay circuit, implemented with a transcriptional repressor expressed in only one state, to ensure that each input pulse results in only one inversion of the DNA segment. Development and optimization of the latch in living cells was driven by mathematical modelling of the recombination reactions and gene expression regulated by the switch. We discuss how *N* latches built with orthogonal site-specific recombination systems could be chained together to form a binary ripple counter that could count to 2*N* − 1.

## INTRODUCTION

Over the past two decades, synthetic biologists have designed and constructed a range of genetic circuits inspired by electronic circuits (1). One type of electronic circuit that has not yet been fully implemented genetically is a counting device. A cell-autonomous genetic counter could be used to keep track of cellular events, such as the number of cell divisions that have occurred in each cell of a population, or the number of exposures to an extracellular event (2). A counter could also be used to step through a programmed series of events, much like the electronic controller of a washing machine, expressing different combinations of genes at each stage.

**Figure 1.**
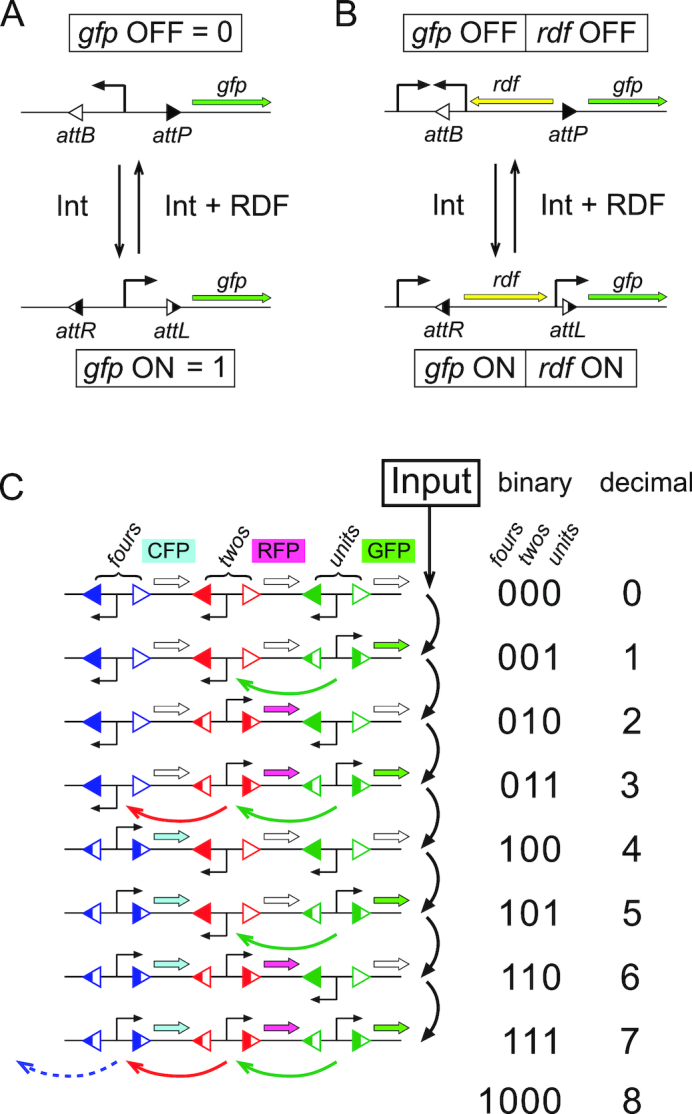
Inversion switches for binary memory and counters. (**A**) The integrase (Int) protein on its own catalyses recombination between DNA sites *attP* and *attB* to create two new sites, *attL* and *attR*. Recombination between *attL* and *attR* only occurs if the recombination directionality factor (RDF) and integrase are both present. If *att* sites are in inverted repeat on either side of a promoter, recombination will reverse the orientation of the promoter and control transcription of a gene outside the invertible DNA segment (e.g. the gene for green fluorescent protein (GFP)). The two states of the inversion switch can be thought of as representing the binary digits 0 and 1. (**B**) Expression of the RDF can be placed under the control of the switch so that is it expressed in the LR state but not the PB state. A pulse of integrase expression should toggle the state of the switch from PB→LR or LR→PB. (**C**) Three inversion switches based on orthogonal integrases can be used to represent any binary number from 0 to 111 (0 to 7 decimal). The state of each switch is output through expression of three different fluorescent proteins, exemplified here as green-, red- and cyan fluorescent proteins (GFP, RFP and CFP). The figure shows the design for a binary ‘ripple’ counter that will step sequentially through all binary numbers between 000 and 111 in response to a repeated input signal. Each input signal (black arrows) toggles the state of the ‘units’ (GFP) switch so that it alternates between PB (0) and LR (1) states. As the units switch changes from 1 to 0, it sends a signal (green arrows) to toggle the ‘twos’ (RFP) switch, which in turn sends a signal (red arrows) to toggle the ‘fours’ (CFP) switch as it changes from 1 to 0.

One way to store numbers in DNA sequences is to use site-specific recombination to flip the orientation of DNA segments (Figure 1A). A previous genetic counter used two site-specific inversion systems to count two repeats of an input signal (3). The first input induced expression of one recombinase, changing the state of the device so that the second input induced the second recombinase. In general, this encoding allows *N* recombinases to record *N* events. A more efficient use of recombinase-mediated DNA inversion for counting would be to encode numbers using the binary system. An invertible DNA segment has two possible orientations, or states, that can be used to represent the binary digits 0 and 1 (4,5). Using multiple invertible DNA segments, each controlled by a different orthogonal recombinase, multiple binary digits can be stored in the DNA (6). Using this encoding, *N* recombinases could be used to count from 0 to 2*^N^* − 1 instead of just to *N*.

The large serine integrases and their recombination directionality factors (RDFs) have been used previously to create switches and logic gates (4,5,7,8), and are ideally suited to creating inversion switches for binary counters (9). Integrase proteins on their own catalyse efficient recombination between two short (∼50 bp) DNA sequences: *attP* and *attB* (10). This produces two new sites (*attL* and *attR*) that are not recombined further by integrase alone (Figure 1A). However, in the presence of the cognate RDF protein, integrase directionality is switched so that *attL* and *attR* recombine efficiently to recreate *attP* and *attB*. If *att* sites are in inverted (head-to-head) repeat, an inversion switch is created that can be repeatedly flipped back and forth between two states by the alternate expression of integrase and integrase plus its RDF (Figure 1A). Over 20 orthogonal serine integrases have been characterized to date (6,11), and many now have identified RDFs.

To make a circuit that can count in binary up to 2^*N*^ − 1, *N* inversion switches can be connected together to form a ‘ripple’ counter (Figure 1C) (12). The units (the right-most digit in the binary number) are recorded by a switch that repeatedly toggles between 0 and 1 each time an input signal is received. The next switch (recording the number of twos) toggles between 0 and 1 every time the first switch changes from 1 to 0. Similarly, the next switch (the fours) is toggled every time the twos switch changes from 1 to 0, and so on. The binary counter therefore requires multiple orthogonal toggle switches, each one flipping between 0 and 1 every time it receives a signal, and passing a signal on to the next each time it makes the transition from 1 to 0. Here, we describe the development of a one-input, synthetic toggle switch based on φC31 integrase-mediated inversion that could form the basis of such a multi-bit ripple counter.

## MATERIALS AND METHODS

### Bacterial strains and growth conditions

Recombination assays were performed in *Escherichia coli* DS941 (AB1157 *recF lacI^q^ lacZΔM15* (13)) or in DS941 Z1. The Z1 construct (14) carries two copies of *lacI^q^* and *tetR* transcribed from P_N25_, and was moved into DS941 by P1 transduction. Bacteria were grown at 37°C in lysogeny broth (LB; 10 g/l tryptone, 5 g/l yeast extract and 10 g/l NaCl). Agar (15 g/l) was added for solid media. Liquid cultures were grown in 150 × 25 mm glass tubes with vented caps, shaking at 225 rpm. Antibiotics were added at the following concentrations where appropriate: ampicillin 100 μg/ml, kanamycin 25 μg/ml, chloramphenicol 25 μg/ml and spectinomycin 50 μg/ml. Transcription from P_BAD_ was induced with 0.2% (w/v) arabinose (15). P_LtetO-1_ was induced with anhydrotetracycline (aTc) at 100 ng/ml in the presence of 0.1 mM MgCl_2_ (14).

### Plasmids

The construction of plasmids used in this study is described in detail in the Supplementary Data. All plasmids were verified by DNA sequencing. Annotated DNA sequences are available in GenBank format in the Supplementary Data.

### *In vivo* switching assays

Switch and integrase expression plasmids were introduced into DS941 or DS941 Z1 sequentially by CaCl_2_ transformation, selecting transformants on LB agar plates with appropriate antibiotics at each stage. Plates contained 0.2% (w/v) glucose and storage time was minimized to reduce recombination due to leaky recombinase expression. Recombination assays were then carried out by one of the methods described below:

#### Overnight induction

A single transformant colony was inoculated into 5 ml of LB containing 0.2% glucose to repress integrase expression, or arabinose and/or aTc to induce recombination. Cultures were incubated overnight (18 h) at 37°C. For repeated cycles, overnight cultures were diluted 1:1000 into fresh media with appropriate inducers and incubated for a further 18 h. Plasmid DNA was prepared from overnight cultures and fluorescence was measured as described below.

#### Continuous time course of induction

To measure the kinetics of recombination *in vivo*, a single transformant colony was first inoculated into 5 ml of LB broth containing appropriate antibiotics and 0.2% glucose, and incubated overnight (18 h) at 37°C. The overnight culture was diluted 1:40 into fresh LB without glucose or inducer, and pre-cultured for 90 min. Arabinose and/or aTc were then added to the exponentially growing cells to induce recombination. Samples (volume adjusted to obtain a constant weight of cell pellet) were removed from the induced culture at different time points after induction. Cells were harvested by centrifugation, flash-frozen in liquid nitrogen and then stored at −20°C prior to plasmid DNA preparation.

#### Pulsed induction time courses of recombination

To obtain a pulse of integrase expression of chosen duration, a single transformant colony was first inoculated into 5 ml of LB broth containing appropriate antibiotics and 0.2% glucose (to repress integrase expression) and incubated overnight (18 h) at 37°C. The overnight culture was diluted 1:40 into fresh LB with antibiotics but without glucose or inducer, and pre-cultured for 90 min (pSWITCH1 and pSWITCH2) or 120 min (pSWITCH3). Arabinose and/or aTc were then added to these exponentially growing cultures to induce recombination. Recombination was stopped at chosen time points after induction by removing 5 μl samples from the induced culture and diluting 1:1000 into 5 ml of fresh LB broth containing appropriate antibiotics and 0.2% glucose. Plasmid DNA was prepared from these overnight cultures, or fluorescence was measured as described below.

Multiple cycles of pulsed operation were carried out exactly as described above, except that a specific induction pulse length was chosen (15–60 min; see figure legends). After each overnight growth in the presence of glucose, 0.5 ml of culture was diluted 1:40 in media without glucose, and inducers were added after 90 or 120 min to start the next cycle.

### Preparation of plasmid DNA

Plasmid DNA was prepared using a QIAprep Spin Miniprep Kit (Qiagen), as described in the manufacturer’s instructions. About 4.5 ml of overnight cell culture was used routinely, but larger volumes (10–20 ml) were used for the early time points in the continuous induction time course experiments.

### Gel electrophoresis and quantification

Gel electrophoresis was in 1.2% agarose gels in TAE buffer (40 mM Tris, 20 mM acetic acid, 1 mM ethylenediaminetetraacetic acid) for 16 h at ∼1.5 V/cm. Gels were stained with 0.5 μg/ml ethidium bromide for at least 40 min and then destained in 1 x TAE for 20 min. Gels were photographed under UV transillumination using a Bio-Rad Gel-Doc, or scanned with a Typhoon FLA900 scanner (GE Healthcare) using the settings for ethidium (532 nm excitation and long pass red emission filter) and are shown in reverse contrast. Band intensities were quantified using Quantity One using rectangle volume mode or Image Quant software using lane peak mode.

### Measurement of bacterial fluorescence

Cells were washed in Phosphate buffered saline (PBS; 8 g NaCl, 0.2 g KCl, 1.44 g Na_2_HPO_4_ and 0.24 g KH_2_PO_4_ per litre) and fixed with formaldehyde prior to fluorescence measurements as follows: 1 ml of cell culture was centrifuged (6000 *g*, 2 min) and washed twice with 1 ml of 0.22 μm-filtered PBS, centrifuging at 6000 *g* for 2 min for each wash. Cells were resuspended thoroughly in 900 μl of filtered PBS, mixed with 100 μl of 40% (v/v) formaldehyde and incubated for 1 min at room temperature to fix. Cells were then washed twice more with 1 ml of PBS, resuspended in 1 ml of PBS and stored at 4°C in the dark for up to 1 week prior to fluorescence measurement.

Single-cell fluorescence was measured by flow cytometry on a FACSAria I Cell sorter. Fixed cells were diluted to ∼10^6^ cells/ml. A total of 30 000 events were acquired using 488 nm laser light with ∼500 events s^−1^ flow rate. The collected data were analysed using Flowjo after gating cells by forward and side scatter.

Fluorescence images of 200 μl samples of PBS-washed cells were acquired in flat-bottomed 96-well plates using a Typhoon FLA900 scanner (GE Healthcare). Settings for GFP were 488 nm laser excitation and the band-pass blue emission filter (530DF20). Settings for RFP were 532 nm laser excitation and the long-pass green emission filter (575 LP).

### Modelling

The mathematical model of the latch circuit describes the intracellular production and decay of all the proteins used to control the latch: ϕC31 integrase, ϕC31 RDF and the *tetR*-encoded tetracycline repressor. Transitions between PB and LR states are modelled using a previously developed set of equations for recombination by ϕC31 integrase, with and without RDF (16). The full description of the model is presented as Supplementary Data. The parameters for recombination reactions were taken from our previously published model (16), rate constants for the degradation of mRNA and transcription from P_LtetO-1_ were taken from elsewhere in the literature (17–19), while other rate constants for the production of integrase, RDF and TetR were fitted to our data (Supplementary Table S1). The system is described by 35 ordinary differential equations and was solved using MATLAB (MathWorks UK, Cambridge). Stochastic segregation of plasmids and changes of fluorescence in dividing cells were also modelled using MATLAB. MATLAB code for all models is available at https://github.com/alex297/model-of-binary-counter-based-on-recombination-with-serine-integrase.

## RESULTS

### Design for a recombinase-based, one-input toggle switch

In this study, we aimed to produce an inversion switch that can be switched between two states, at each repeat of a single input signal. The response of the switch should depend on its state. If the switch is OFF, an input pulse should toggle it ON; if the switch is ON, an input should toggle it OFF. In our design, the two states of the switch are represented by the two possible orientations of an invertible DNA segment flanked by a pair of recombination sites: *attP* and *attB* in one state, *attL* and *attR* in the other (Figure 1A). Throughout the rest of the text, we refer to these two states as ‘PB’ and ‘LR’, respectively. Expression of integrase on its own changes the state of the switch from PB to LR (PB→LR), while co-expression of integrase and RDF changes the state back from LR to PB (LR→PB). A promoter within the invertible DNA segment can be used to transcribe genes outside of the switch in a state-dependent manner (Figure 1A). Here, we use changes in expression of fluorescent proteins (GFP or RFP) to report on the state of the switch, and changes in position of restriction enzyme cleavage sites within the invertible segment to quantify the amount of DNA in the two possible states.

In our design for a one-input toggle switch, expression of the RDF gene is placed under the control of the switch (Figure 1B). In the PB state, RDF is not expressed and a pulse of integrase stimulates recombination between *attP* and *attB* to give the LR state. In the LR state, RDF is expressed and a pulse of integrase should lead to recombination between *attL* and *attR* to restore the PB state.

### Characterizing the components of the inversion switch

**Figure 2.**
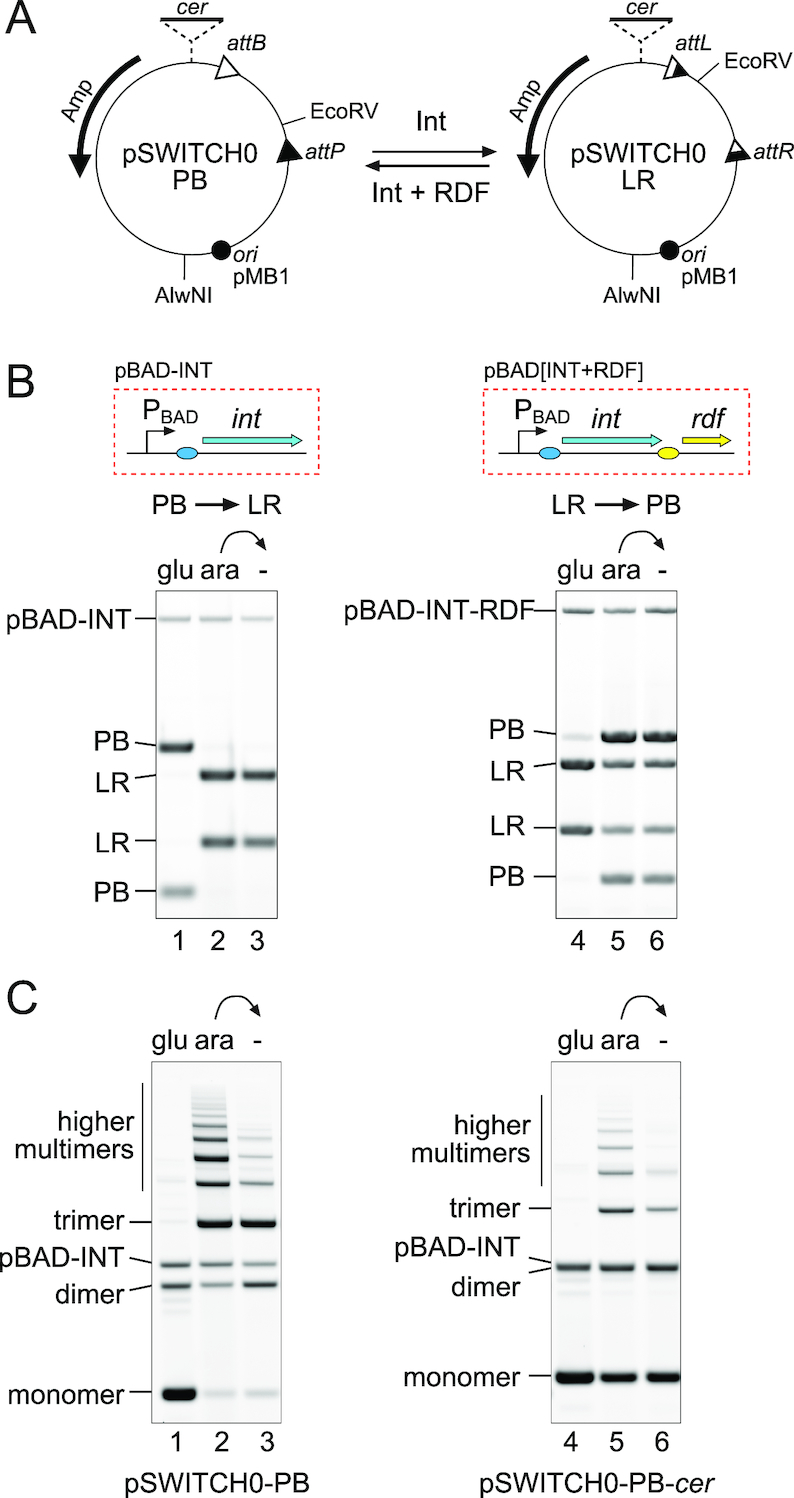
Directional recombination on high copy-number plasmids. (**A**) Diagrams of high copy-number switch plasmids containing an invertible DNA segment in the PB (pSWITCH0-PB) or the LR state (pSWITCH0-LR). (**B**) These plasmids were introduced into *Escherichia coli* DS941 together with a plasmid expressing either φC31 integrase (pBAD-INT; left panel) or integrase with its RDF (pBAD[INT+RDF]; right panel) from the arabinose-inducible P_BAD_ promoter. Cells were grown overnight in LB containing 0.2% glucose to repress expression from P_BAD_ (lanes 1 and 4), or overnight with 0.2% arabinose to induce expression from P_BAD_ (lanes 2 and 5). The cells that had been grown overnight with arabinose were subsequently diluted 1:1000 and grown overnight again without inducer (lanes 3 and 6). Plasmid DNA was purified and cut with AlwNI and EcoRV. (**C**) The left panel (lanes 1–3) shows the same samples as shown in the left panel of (B), run on an agarose gel without prior restriction digestion, to reveal plasmid multimers produced by intermolecular recombination. A multimer resolution site (*cer*) was added to pSWITCH0 at the position indicated in (A) (dotted lines) to create pSWITCH0-*cer*. DS941 containing pSWITCH0-PB-*cer* and pBAD-INT was grown with or without arabinose induction as in (B) and analysed by gel electrophoresis without restriction digestion (lanes 4–6).

We chose the well-characterized φC31 integrase as the basis of our one-input toggle switch. This recombinase has been extensively used for transgene integration and for gene assembly applications (9,20–22). To show that φC31 integrase and its RDF (φC31 gp3 (23)) can function efficiently in an inversion system in *Escherichia coli*, we constructed a high copy-number plasmid (pSWITCH0-PB; Figure 2A) that contains *attP* and *attB* in inverted repeat, and a separate plasmid (pBAD-INT; Figure 2B) that contains the φC31 *int* gene under the control of the arabinose-inducible P_BAD_ promoter (15). These plasmids were co-introduced into the *Escherichia coli* strain DS941, and DNA inversion was monitored by restriction digestion. In the presence of glucose, integrase expression was repressed and no inversion occurred (Figure 2B, lane 1). When arabinose was added to the cells, the restriction pattern of pSWITCH0-PB changed from PB→LR (Figure 2B, lane 2). Due to the unidirectional nature of integrase recombination, once the state of the switch had changed to LR, it remained stably in this state in the presence or absence of further integrase expression (Figure 2B, lane 3).

We next investigated whether the switch could be flipped from LR→PB by expression of φC31 integrase together with its RDF. We isolated pSWITCH0 in the LR state (pSWITCH0-LR; Figure 2A) and expressed integrase with its RDF as a bicistronic unit from the P_PAD_ promoter on pBAD[INT+RDF] (Figure 2B). Addition of arabinose to DS941 cells containing both these plasmids induced integrase and RDF expression and changed ∼70% of the DNA from LR→PB. Our previous *in vitro* experiments demonstrated that stoichiometric amounts of RDF are required to activate ϕC31 integrase for efficient LR→PB recombination (16). The less than complete conversion of LR→PB seen in Figure 2B might therefore be due to insufficient expression of RDF relative to integrase in this experiment. In order to optimize both the ‘forward’ PB→LR and the ‘reverse’ LR→PB reactions, we assessed different expression levels of integrase (by altering its translation initiation rate) and RDF (by placing it on plasmids of different copy-number) within the cell (Supplementary Figure S1). A threshold level of integrase expression was required for efficient PB→LR conversion *in vivo*. This threshold depended on the copy-number of the plasmid carrying the inversion switch; higher copy-number switch plasmids required higher levels of integrase expression for efficient recombination (Supplementary Figure S1). Conversely, LR→PB recombination was less efficient at the highest integrase expression levels, presumably because RDF levels were insufficient to saturate integrase at these higher levels (Supplementary Figure S1).

Although recombination of pSWITCH0 from PB→LR was highly efficient *in vivo*, with >95% of the DNA changing to LR after integrase expression (Figure 2B, lane 2), analysis of uncut plasmid DNA revealed that much of the product was in the form of plasmid multimers (Figure 2C, lane 2). These multimers are produced when recombination occurs between *attP* on one plasmid copy and *attB* on another (i.e. recombination is intermolecular rather than intramolecular; Supplementary Figure S2). Although this yields products with the expected LR restriction pattern, plasmid multimers are inherited less stably than are monomers at cell division (24). Subsequent rounds of LR→PB and PB→LR recombination (as required for counting applications) will likely also be intermolecular, leading to ever larger plasmid multimers and to increased plasmid loss. To counteract this, a *cer* multimer resolution site was added outside the invertible segment in pSWITCH0-PB to form pSWITCH0-PB-*cer*. In this plasmid, recombination at *cer* by the *Escherichia coli* Xer system should monomerize any multimers produced by integrase (Supplementary Figure S2). As expected, after integrase induction *in vivo*, pSWITCH0-PB-*cer* remained more monomeric than did pSWITCH0-PB (Figure 2C, compare lanes 1–3 with 4–6).

After these initial investigations, we settled on a relatively low copy-number pSC101-based plasmid vector containing a *cer* site to carry inversion switches, and the medium copy-number p15a-based plasmid pBAD-INT to express integrase under the control of arabinose.

### Kinetics of recombination

To determine how fast recombination takes place after inducing integrase expression, DS941 cells carrying pBAD-INT-106 (with a reduced level of integrase expression) and the low copy-number inversion switch plasmid (pSWITCH0_LC_-PB; Supplementary Figure S3A) were grown to mid-exponential phase. Integrase expression was induced by the addition of arabinose, and cells were frozen in liquid nitrogen to stop recombination at different time points. Plasmid DNA was isolated and analysed by restriction digestion. Recombination was detectable 10 min after arabinose addition and was essentially complete by the 60-min time point (Supplementary Figure S3B).

**Figure 3.**
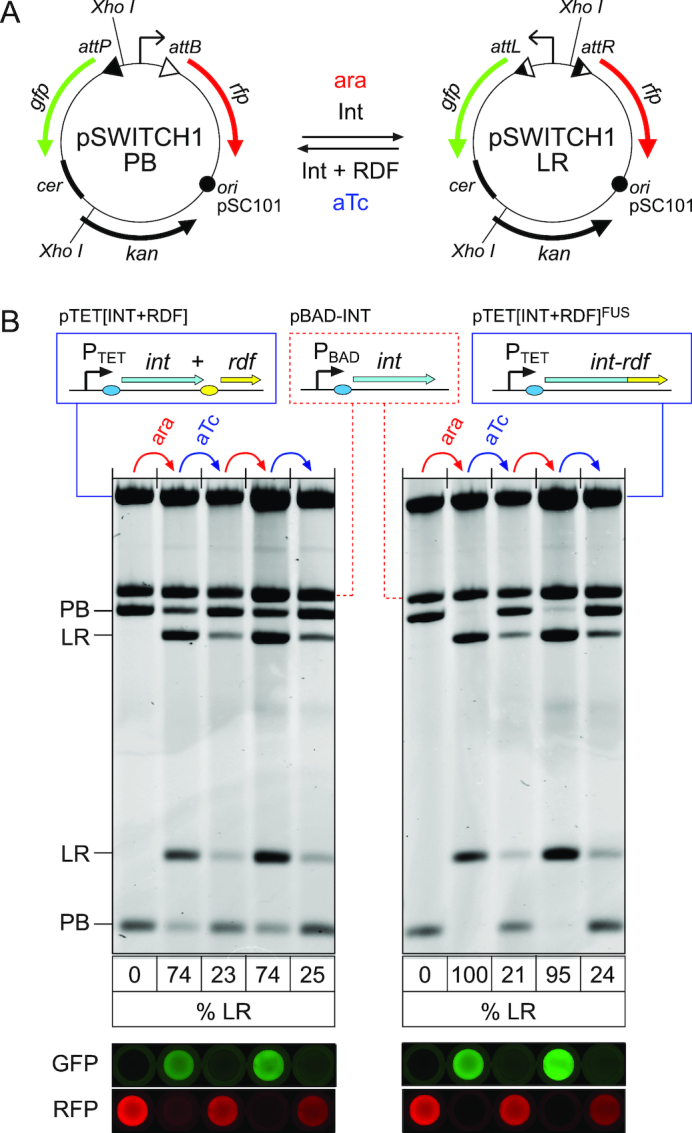
Repeated operation of a low copy-number inversion switch by inducible expression of integrase and RDF. (**A**) pSWITCH1 has a low copy-number pSC101 origin and a *cer* site and carries φC31 *att* sites flanking a constitutively active promoter (arrow). RFP is expressed in the PB state, while GFP is expressed in the LR state. (**B**) pSWITCH1-PB was introduced into *Escherichia coli* DS941 cells together with pBAD-INT and either pTET[INT+RDF] expressing integrase and RDF as two separate proteins (left), or pTET[INT+RDF]^FUS^ expressing the Int-RDF fusion protein (right) under the control of the aTc inducible P_LtetO-1_ promoter (P_TET_). Cells were grown overnight in 0.2% glucose (lane 1 on each gel). Cultures were diluted 1:40 into fresh LB and grown for 90 min. Arabinose was then added, and cells were grown for a further 30 min before 1:1000 dilution and overnight growth in fresh media containing 0.2% glucose. This treatment constitutes an arabinose pulse (ara). This was followed by similar aTc, then arabinose, then aTc pulses. DNA was isolated from each overnight culture, cleaved with XhoI and analysed by agarose gel electrophoresis. The percentage of pSWITCH1 in the LR state is shown beneath each lane. GFP and RFP fluorescence scans of 200 μl of cell suspension in 96-well plates after each overnight culture are shown aligned with the corresponding lanes of the gels.

Our next-generation switch (SWITCH-1) contained genes for RFP and GFP on either side of an invertible DNA segment. This invertible segment contains a constitutive promoter oriented towards RFP in the PB state, but towards GFP in the LR state. The complete module was placed on a low copy-number pSC101-based plasmid with a *cer* site to give pSWITCH1-PB (Figure 3A).

A method to deliver short pulses of integrase expression was then tested. *Escherichia coli* DS941 containing pBAD-INT and pSWITCH1-PB was grown to mid-exponential phase and arabinose was then added to induce integrase expression. At different time points after induction, cells were diluted 1000-fold into fresh medium containing glucose without arabinose to stop further integrase expression. Cells were then grown overnight to stationary phase and plasmid DNA was isolated for restriction analysis (Supplementary Figure S4B). Very little recombination (∼7%) was observed after a 5-min pulse of arabinose delivered in this way, demonstrating that dilution effectively stops the induction. In contrast, a 30-min pulse of arabinose was sufficient to give ∼80% recombination, and recombination was essentially complete after a 60-min pulse. A similar experiment was carried out using pSWITCH1-LR and pBAD[INT+RDF] (Supplementary Figure S4C). Recombination started shortly after induction of integrase and RDF with arabinose, and went to ∼80% completion after a 60-min induction pulse.

### Repeated operation of the inversion switch

We next investigated whether pSWITCH1 could be flipped repeatedly between PB and LR states by alternate expression of φC31 integrase (for PB→LR recombination) and integrase together with RDF (for LR→PB). Integrase was expressed from pBAD-INT (as above), and integrase plus RDF were expressed from the anhydrotetracycline (aTc)-inducible P_LtetO-1_ promoter (14) on a third plasmid (pTET[INT+RDF]; Figure 3B). All three plasmids were co-introduced into *Escherichia coli* in the absence of arabinose and aTc to yield cells that contained DNA exclusively in the PB state and (Figure 3B, lane 1). A 30-min pulse of arabinose changed ∼75% of the DNA to the LR state (Figure 3B, lane 2). A subsequent pulse of aTc changed ∼75% of the DNA back to PB (Figure 3B, lane 3). The switch between PB and LR states continued to operate over two further cycles of arabinose and aTc induction, with similar efficiencies each time (Figure 3B, lanes 4–5).

To test whether DNA inversion regulates expression of GFP and RFP effectively in pSWITCH1, we examined fluorescence levels after each induction (Figure 3B). In the initial PB state, cells expressed RFP but not GFP. As expected, GFP fluorescence was high and RFP was low after each arabinose pulse, whereas RFP was high and GFP was low after each aTc pulse.

### Improved switching using an integrase-RDF fusion

In the experiment reported above with pSWITCH1, induction of integrase with arabinose gave only ∼74% PB→LR recombination (Figure 3B left panel), in contrast to the nearly complete PB→LR recombination seen in earlier experiments (Figure 2B). We hypothesized that this incomplete PB→LR recombination might be due to leaky expression of RDF from pTET[INT+RDF] in the absence of aTc. We hypothesized further that this leaky expression might come from transcription initiating within the integrase gene, and that this could be eliminated by the use of a recently reported fusion between φC31 integrase and its RDF (25), because the RDF gene lacks its own translation initiation signals in this fusion.

The separate integrase and RDF genes on pTET[INT+RDF] were replaced with the integrase-RDF fusion to yield pTET[INT+RDF]^FUS^ (Figure 3B). *Escherichia coli* DS941 was co-transformed with pTET[INT+RDF]^FUS^, pBAD-INT and pSWITCH1-PB, and switching was tested over multiple cycles (Figure 3B, right panel). Integrase induction with arabinose gave highly efficient PB→LR conversion (95–100%), consistent with reduced leaky expression of RDF in the absence of aTc, while induction of the fusion protein with aTc gave 75–80% conversion back to PB.

Although use of the Int-RDF fusion improved the performance of SWITCH-1, and demonstrated that tight regulation of RDF expression is essential for directional recombination, the design of our one-input toggle switch requires independent regulation of RDF expression by the switch, while integrase is regulated by the input signal. We therefore continued the development of our one-input toggle switch using separate integrase and RDF genes.

### Placing RDF expression under the control of the switch

**Figure 4.**
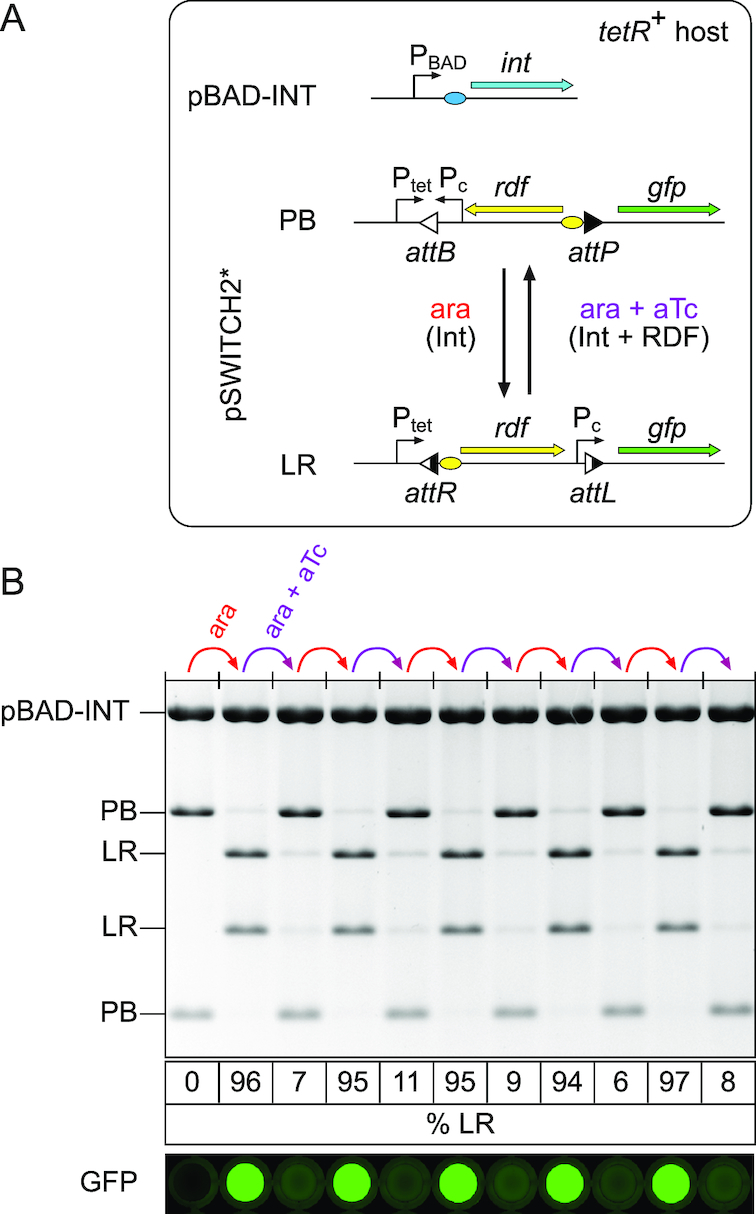
Repeated operation of a switch with state-dependent expression of RDF. (**A**) *Escherichia coli* DS941 Z1, which has a chromosomal copy of the tetracycline repressor gene, was co-transformed with a plasmid expressing φC31 integrase from the P_BAD_ promoter (pBAD-INT) and pSWITCH2*. φC31 RDF is expressed from P_LtetO-1_ (P_TET_) and an optimized RBS (yellow oval) on pSWITCH2*, but only when aTc is present and the switch is in the LR state. In the PB state, arabinose induces expression of integrase leading to the conversion of PB→LR. In the LR state, addition of arabinose and aTc induces integrase and RDF, converting LR→PB. (**B**) Starting from the PB state, DS941 Z1 containing pSWITCH2* and pBAD-INT was subjected to five cycles of induction first with arabinose and then with arabinose and aTc. For each induction, cells from the previous stage were diluted 1:1000 and then grown overnight to stationary phase with either 0.2% arabinose or 0.2% arabinose plus 100 ng/ml aTc. Plasmid DNA was isolated and cleaved with NheI before agarose gel electrophoresis. The percentage of pSWITCH2* DNA in the LR state is shown below each lane. GFP fluorescence scans of cell cultures at each stage are shown aligned with the corresponding lanes of the gel above.

In order to put RDF expression under the control of the switch, a promoterless copy of the RDF gene was placed in the invertible segment, and the aTc-inducible P_LtetO-1_ promoter was placed just outside *attP* and *attB* to produce pSWITCH-2 (Figure 4A). When the invertible segment is in the LR configuration, RDF is correctly orientated to be transcribed from P_LtetO-1_, whereas in the PB state the RDF gene will not be transcribed (Figure 4A). A constitutive promoter downstream of the RDF gene in the invertible segment drives GFP transcription only when the switch is in the LR state. P_LtetO-1_ was used to express RDF in pSWITCH-2, as this promoter will be constitutively active in *Escherichia coli* strains (such as DS941) that lack the tetracycline repressor gene (*tetR*), as required for our one-input switch. However, in *Escherichia coli* strains that express *tetR* (e.g. DS941 Z1 (14)), RDF expression should depend both on the state of the switch and on the presence of aTc, allowing for easy debugging of the switch.

Recombination of pSWITCH-2 was first tested in the presence of *tetR*. *Escherichia coli* DS941 Z1 cells containing pBAD-INT and pSWITCH2-PB were subjected to different pulse lengths of arabinose. Recombination PB→LR was first apparent after a 10-min pulse of arabinose, and longer pulses (20 min to 2 h) converted ∼80% of the DNA to LR (Supplementary Figure S5B). To test recombination in the other direction, DS941 Z1 containing pBAD-INT and pSWITCH2-LR was induced with arabinose and aTc. Recombination LR→PB started more slowly than did PB→LR, but was essentially complete (>90%) after pulses of 30 min or longer (Supplementary Figure S5C).

We hypothesized that leaky RDF expression from SWITCH2 in the absence of aTc might account for the less than complete PB→LR recombination (∼80%; Supplementary Figure S5B). To lower this leakage and improve PB→LR recombination, a random library of ribosome-binding sites was inserted upstream of the RDF gene in pSWITCH2-LR (Supplementary Figure S6). Mutants that retained good performance in the LR→PB direction were picked as colonies that had low GFP fluorescence following a pulse of arabinose plus aTc, and were then screened for high GFP fluorescence after a pulse of arabinose. After retesting several candidates (Supplementary Figure S6B and C), we picked an improved pSWITCH-2 (pSWITCH-2*) with reduced ribosome-binding site strength for RDF (Supplementary Figure S6D) that recombined efficiently in both the PB→LR (∼95%) and LR→PB (>90%) directions.

We next tested the operation of pSWITCH-2* with pBAD-INT in DS941 Z1 over multiple set-reset cycles, using alternate pulses of arabinose to express integrase, followed by arabinose and aTc to express integrase and RDF. Plasmid DNA was checked by restriction digestion, and population fluorescence was measured after each pulse (Figure 4B). pSWITCH2* operated efficiently for five complete set-reset cycles, with no decrease in efficiency, as measured by examining the state of the DNA or cell fluorescence. Approximately 95% of the DNA was in the LR state after each arabinose pulse, and >89% of the DNA was in the PB state after each arabinose-aTc pulse (Figure 4B).

### Testing for one-input operation

In a strain that lacks the tetracycline repressor, P_LtetO-1_ will be constitutively active and RDF expression will be controlled solely by the state of the switch. If our original design concept is correct, the system should therefore act as a one-input toggle switch, switching PB→LR and LR→PB after each arabinose pulse. We therefore tested switching of pSWITCH2*-PB and pSWITCH2*-LR in DS941 with pBAD-INT, using arabinose pulses of different durations. Starting from pSWITCH2*-LR, arabinose pulses from 10 min to 2 h gave efficient (∼85%) switching to PB (Supplementary Figure S7C), demonstrating that RDF is expressed from P_LtetO-1_ in this strain. However, starting from pSWITCH2*-PB, recombination was very poor (<20% conversion to LR) for all arabinose pulse lengths (Supplementary Figure S7B).

### Mathematical modelling of the single-input toggle switch

**Figure 5.**
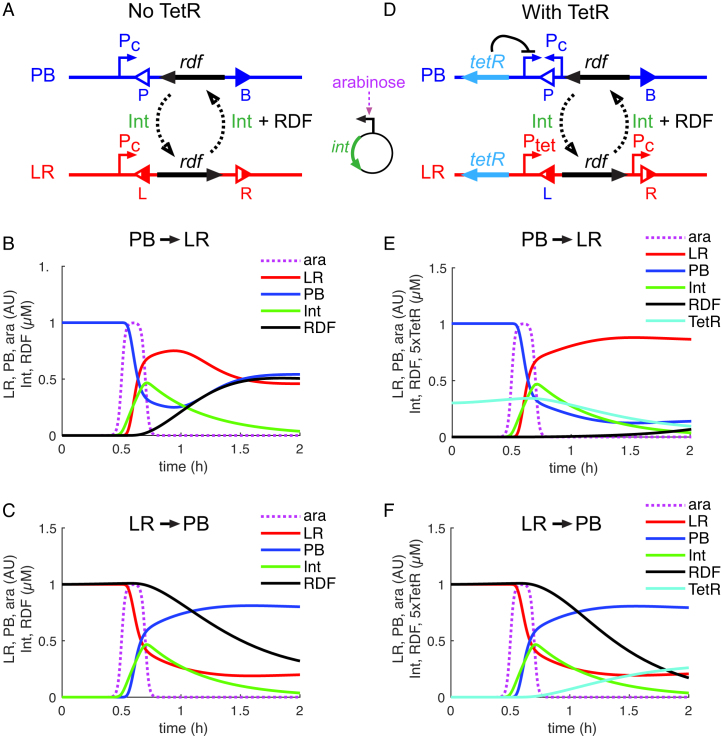
Modelling of one-input switches. (**A** and **D**) Diagrams showing the genetic circuits for one-input switches without (A) or with (D) the TetR-based delay circuit. In (A), the RDF is expressed from a constitutively active promoter (P_c_) only in the LR state. In (D), RDF is expressed from a TetR-repressed promoter (P_tet_) in the LR state, and TetR is expressed from a constitutively active promoter (P_c_) in the PB state. The input signal is provided by short pulses of arabinose that induce expression of integrase protein (Int) from a separate plasmid (shown between A and D). Int (with or without RDF) provides transitions between the PB (blue) and LR (red) states of the switch DNA. In (D), expression of TetR in the PB state delays RDF expression during the PB→LR transition. (**B** and **C**) Modelled kinetics of the PB→LR (B) and LR→PB (C) transitions for the switch without TetR. (**E** and **F**) Modelled kinetics of the PB→LR (E) and LR→PB (F) transitions for the switch with TetR controlled by the switch. Graphs show the relative amounts (arbitrary units; AU) of LR (red), PB (blue), arabinose (purple) and the concentrations (μM) of Int (green), RDF (black) and TetR (cyan). A single 12-min arabinose pulse was given at time point 0.5 h.

To analyse the reasons for the failure of our initial design and potential ways to improve it, we built a mathematical model for its operation (Figure 5A–C). The model is based on ordinary differential equations (ODEs), describing intracellular production and degradation of integrase and RDF, and the resulting recombination reactions. The full description of the model and ODEs is presented as Supplementary Data. Briefly, the production of integrase is described through its induction by periodic (daily) arabinose pulses. RDF is expressed from a constitutive promoter (to model P_LtetO-1_ in DS941) only in the LR state (Figure 5A), so the rate of RDF production is proportional to the concentration of LR DNA. The recombination reactions with or without RDF are described by a previously developed set of equations (Supplementary Figure S8; (16)).

The model illustrates how switching of just a small proportion of DNA PB→LR in the absence of TetR leads to rapid RDF production that inhibits further PB→LR recombination and allows the reverse LR→PB reaction to start (Figure 5B). In contrast, LR→PB recombination is relatively efficient because RDF persists throughout the integrase expression pulse (Figure 5C).

### Introducing a delay in RDF expression gives efficient one-input operation

To create a circuit that can complete the PB→LR transition, we reasoned that RDF expression from plasmid molecules that have switched to LR should be delayed until after the end of the integrase pulse. To do this, we added TetR inhibition of the promoter transcribing RDF to the model. We also linked TetR production to the state of the switch, by placing the *tetR* gene outside the invertible DNA segment so that it is expressed from the constitutive promoter in the invertible segment only in the PB state (Figure 5D). Our modelling demonstrated that this new switch design should efficiently switch from PB→LR and from LR→PB after each pulse of arabinose-induced integrase expression (Figure 5E and F), as described below.

In the PB state, the constitutive promoter within the invertible segment directs transcription towards *tetR*, and the RDF gene is in the wrong orientation to be expressed from the TetR-regulated promoter P_LtetO-1_ (Figure 5D). Thus, the concentration of TetR protein is high and that of RDF is low. Induction of integrase switches the invertible segment to LR, placing the RDF gene in the correct orientation to be expressed from P_LtetO-1_. However, P_LtetO-1_ is repressed by TetR, so RDF transcription is delayed until TetR is degraded or diluted by cell growth, allowing near complete recombination from PB→LR during the pulse of integrase expression (Figure 5E).

In the LR state, TetR is not expressed, and the RDF gene is in the correct orientation to be transcribed from the de-repressed P_LtetO-1_ (Figures 5B). Therefore, high levels of RDF are present, and integrase induction leads to efficient switching LR→PB. TetR expression starts as switching begins, repressing further RDF production. However, RDF produced prior to switching persists in the cell throughout the duration of the integrase pulse, and is sufficient for near complete LR→PB recombination (Figure 5F).

**Figure 6.**
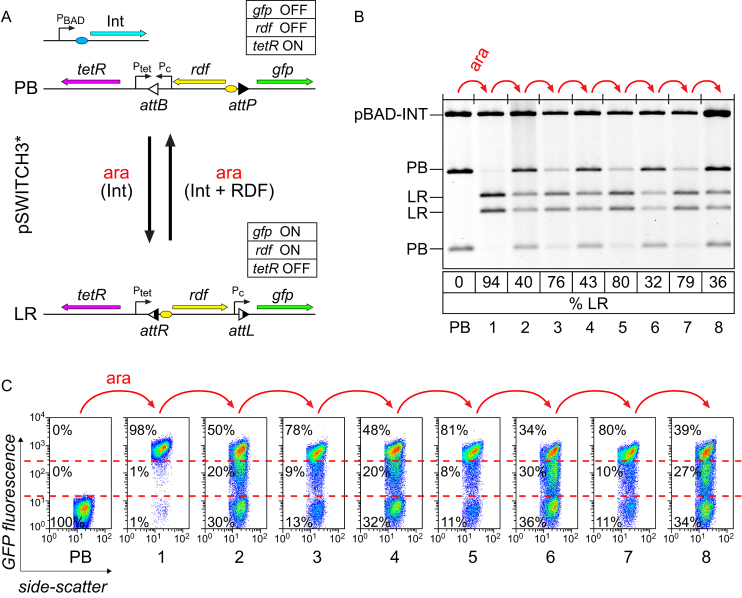
Repeated operation of the pSWITCH3* single-input binary counting module. (**A**) The state-based switch (pSWITCH2) was converted to a single-input binary counting module pSWITCH3 by adding a delay circuit consisting of the *tetR* gene expressed from a constitutive promoter only in the PB state. The ribosome-binding site for the *rdf* gene was then optimized for switching in both directions to give pSWITCH3*. (**B** and **C**) Operation of pSWITCH3* over eight cycles of pulsed integrase expression starting from the PB state. For each cycle, exponentially growing cells were exposed to 0.2% arabinose for 15 min to induce integrase expression, followed by 1:1000 dilution into media containing 0.2% glucose to repress further expression. (B) Plasmid DNA was purified and analysed by restriction digestion and agarose gel electrophoresis. The percentage of DNA in the LR state is shown below in each lane. (C) GFP fluorescence of approximately 30 000 cells was measured by flow cytometry after each overnight culture. Percentages of cells in high, intermediate and low fluorescence states are shown on the plots, with gates shown as red dashed lines.

To construct the genetic circuit shown in Figure 5D, we added the *tetR* gene outside of the invertible DNA region in pSWITCH2, giving pSWITCH3 (Figure 6A). As in the model, the constitutive promoter within the invertible segment drives expression of TetR in the PB state, while the RDF gene is transcribed from the TetR-repressed promoter outside the invertible segment only in the LR state. PB→LR and LR→PB operation of SWITCH3 was tested using arabinose pulses of different lengths. Starting from pSWITCH3-PB, switching to LR was efficient over a wide range of pulse lengths; 80–85% recombination was observed after pulses ranging from 10 min to 2 h (Supplementary Figure S9B). Starting from pSWITCH3-LR, switching to PB was slightly less efficient; ∼70% recombination after arabinose pulses of 10–30 min, and ≤65% with longer integrase expression times (60 or 120 min; Supplementary Figure S9C), suggesting that the RDF concentration starts to decrease towards the end of these longer pulses.

Our modelling showed that the ratio of TetR:RDF expression levels is critical for efficient switch operation. With high expression of RDF relative to TetR, RDF accumulates and the switch gets stuck in a mostly PB state after multiple cycles (Supplementary Figure S10A). Conversely, high expression of TetR relative to RDF traps the switch in the LR state (Supplementary Figure S10B). According to the model, an optimum ratio of RDF:TetR expression levels can be found, at which transitions in both directions (PB→LR and LR→PB) are favourable after each arabinose pulse, and switching continues over multiple cycles of induction (Supplementary Figures S10C and S11).

To improve the performance of pSWITCH3, we therefore optimized the ratio of RDF:TetR expression by randomizing the ribosome-binding sites for the RDF gene and selecting for improved PB→LR and LR→PB switching (Supplementary Figure S12A). Operation of the optimized pSWITCH3*-PB plasmid was tested over eight rounds of switching, using 15-min pulses of arabinose to induce integrase expression from pBAD-INT. Cells were diluted 1000-fold into media containing glucose to end each arabinose pulse, and then grown for ∼10 generations prior to analysis. The state of plasmid DNA was assayed by gel electrophoresis, and the fluorescence of individual cells was monitored by flow cytometry after each treatment with arabinose and outgrowth. The initial PB→LR transition was highly efficient, converting 94% of the DNA to LR and 98% of cells to a high GFP state (Figure 6B and C). The second (LR→PB) transition was less efficient, converting 60% of the DNA back to PB and returning only ∼30% of cells to low GFP (Figure 6B and C). However, a substantial proportion of cells (∼20%) had an intermediate level of fluorescence after this second arabinose pulse, and after the next arabinose pulse most cells (78%) returned to high fluorescence. The population continued to alternate between two different states over the next six cycles of arabinose induction. In one state, ∼80% of cells had high GFP fluorescence and ∼80% of the DNA was LR (Figure 6B and C, pulses 3,5,7), while the other state contained a heterogeneous mixture of cells with low, intermediate and high GFP, with ∼60% of the DNA in the PB state (Figure 6B and C, pulses 2,4,6,8).

The same phenomenon was observed when the experiment started from pSWITCH3*-LR (Supplementary Figure S12B and C). After the first arabinose pulse, nearly 90% of the DNA switched to PB, but only ∼50% of the cells had low fluorescence, while ∼28% had an intermediate level of fluorescence. After the second arabinose pulse, 90% of cells had high fluorescence, and most of the DNA (87%) was LR (Supplementary Figure S12C, pulse 2). Over the next six arabinose pulses analysed, DNA alternated between 75–85% LR and 60–70% PB, and cells alternated between mainly (∼80%) high, and a mixture of low, intermediate and high, fluorescence levels.

The pSWITCH3* plasmid is maintained at ∼20 copies per cell in fast growing cultures (26,27), and this explains the mixed fluorescence levels of cells after LR→PB recombination. Incomplete LR→PB recombination produces individual cells that contain mixtures of plasmids in PB and LR states (e.g. 80% recombination will yield a cell with 16 PB and 4 LR plasmid copies). Over the next 10 generations, random plasmid replication and segregation at cell division yield cells that contain DNA solely in the PB (low GFP) state, cells that retain a mixture of DNA in both states (intermediate GFP) and a small number of cells that contain only LR DNA (high GFP). However, the population continues to cycle between two states over multiple cycles because cells with mixtures of plasmids in both states produce enough TetR from plasmids in the PB state to repress RDF production from plasmids in the LR state, allowing efficient PB→LR recombination at the next arabinose pulse.

### The effect of plasmid segregation

**Figure 7.**
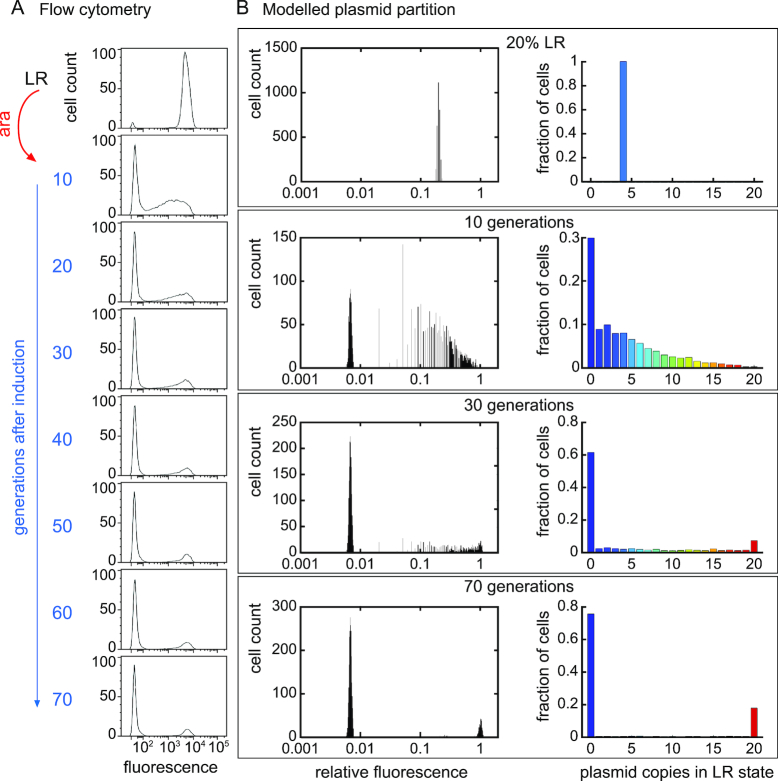
Segregation of pSWITCH3* in PB and LR states. (**A**) Cells containing pBAD-INT and pSWITCH3* in the LR state were subjected to a pulse of arabinose to induce switching to the PB state. Cells were diluted 1000-fold and then grown to stationary phase (10 generations) a total of seven times. At each stage, GFP fluorescence of ∼30 000 individual cells was measured by flow cytometry. (**B** and **C**) Stochastic simulation of cell fluorescence and plasmid segregation over 70 generations. The simulation was started with a population of 3000 cells, all with 16 PB plasmid copies and 4 LR copies (20% LR). In the model, plasmids replicate and then are randomly distributed between daughter cells at cell division. Each cell produces GFP at a rate proportional to the LR copy-number and inherits half the GFP content of its mother cell. The left panels show simulated flow cytometry plots at 0, 10, 30 and 70 generations. The number of cells in each fluorescence bin is plotted. Right panels show the distribution of LR copy-number in cells at 0, 10, 30 and 70 generations.

To test the effect of plasmid segregation on the switch, we subjected DS941 containing pBAD-INT and pSWITCH3*-LR to a single arabinose pulse, and then grew for a further 70 generations without arabinose. Cells were monitored by flow cytometry every 10 generations, for a total of 70 generations (Figure 7A). About 10 generations after the arabinose pulse, 44% of the cells had low fluorescence, ∼29% had intermediate fluorescence and ∼27% remained highly fluorescent (similar to our previous observations; compare Figure 7A with Supplementary Figure S12C). Cells with intermediate GFP fluorescence were lost over further generations of cell growth so that after 70 generations ∼80% of cells had low fluorescence, ∼20% of cells had high fluorescence and almost no cells remained with intermediate fluorescence (Figure 7A).

To show that this behaviour is consistent with random plasmid segregation at cell division, we built a stochastic model for plasmid segregation and cellular fluorescence. The model was started with 3000 cells, each containing 16 PB and 4 LR plasmid copies (20%LR; Figure 7B), as might be expected just after an LR→PB transition. After 10 generations, 30% of cells had only PB plasmid copies and low fluorescence, while the remaining cells had from 1 to 20 LR copies and intermediate to high fluorescence. By 70 generations in the model, ∼80% of the cells had only PB plasmid copies and low fluorescence, while ∼20% of cells had only LR plasmids and high fluorescence (Figure 7B) just as seen in our experimental results (Figure 7A). When the model was initiated with different ratios of PB:LR plasmids in every cell, the proportions of cells with only PB, or only LR DNA after 70 generations accurately reflected the original ratio of PB:LR DNA in the starting cells (Figure 7B and Supplementary Figure S13), as expected if there is no selective advantage for plasmids in the PB or LR states. Thus, the proportion of cells with high or low fluorescence after 70 generations can be used as an accurate measure of the original proportion of DNA in the two states just after recombination.

**Figure 8.**
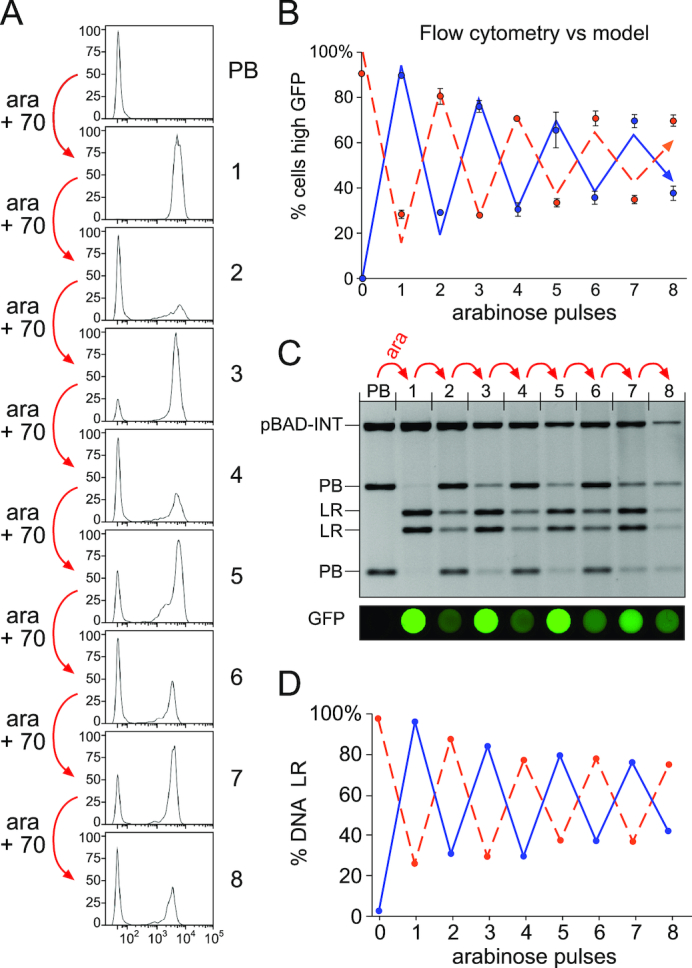
Repeated operation of pSWITCH3* allowing 70 generations for plasmid segregation between arabinose pulses. (**A**) Cells containing pBAD-INT and pSWITCH3*-PB were exposed to eight arabinose pulses, each pulse followed by 70 generations to allow almost complete plasmid segregation. Histograms show fluorescence of ∼30 000 cells measured by flow cytometry after each pulse and 70 generations of growth. (**B**) Graph showing results of three repeats of the same experiment, starting from either pSWITCH3*-PB (blue circles) or pSWITCH3*-LR (orange circles). The percentage of cells with high GFP fluorescence is plotted. Values are the average from three replicates, and error bars represent the standard deviation. Lines show predictions from a model in which 95% of all cells in the PB state change to LR, and 85% of all cells in the LR state change to PB at each pulse. (**C**) Plasmid DNA was isolated from the samples shown in (A), cleaved with SpeI and analysed by agarose gel electrophoresis. Fluorescence scans of 200 μl of cell culture at each stage are shown aligned with the corresponding lanes of the gel above. (**D**) Quantitation of DNA in PB and LR states, starting from either PB (blue line) or LR (orange dashed line).

In the experiments reported in the previous section, cells containing pSWITCH3* switched between two states in response to arabinose signals repeated every ∼10 generations. Practical applications of pSWITCH3* for binary counting might require stable memory of state for many generations between signals. To test whether this is achievable, we performed an experiment with eight arabinose pulses, each followed by 70 generations of cell growth. This will allow cells to segregate into distinct PB and LR states before the next arabinose signal. The fluorescence of individual cells was measured by flow cytometry after each cycle. The results of a typical experiment are shown in Figure 8A, and the average fluorescence from three repeats, starting from either PB or LR, is plotted on Figure 8B. As expected, only a small proportion of cells had intermediate fluorescence levels after each cycle (Figure 8A). The cells switched repeatedly between mainly high and mainly low fluorescence states, but the amplitude of this oscillation gradually decreased over the course of the experiment (Figure 8B). The fraction of plasmid DNA in PB and LR states showed a similar pattern of damped oscillation (Figure 8C and D). These results are consistent with ∼95% of PB cells converting to LR, and ∼85% LR cells converting to PB after each cycle of arabinose treatment and plasmid segregation (Figure 8B, blue and orange lines). After many cycles of switching at these efficiencies, the population will eventually reach a steady state (∼52%LR, ∼48% PB) in which most (∼90%) cells change state at each signal, but the number of cells changing PB→LR is equal to the number changing LR→PB.

## DISCUSSION

We have used φC31 integrase and its RDF to produce a fully functional, synthetic, single-input toggle switch, the first time this has been achieved to our knowledge. RDF expression was placed under the control of the switch so that directional switching occurred every time an input signal produced a pulse of integrase expression. The switch was toggled repeatedly between two different states for multiple cycles, and in the absence of any signal stably retained its state for many generations.

There are two published computer models for single-input recombinase-based toggle switches that we know of. Both use very different genetic circuitry from our latch. One uses two DNA inversion switches for each latch; the first inversion switch stores the state of the device in the absence of an input, while the second stores the previous state during the course of an input pulse to ensure that each input leads to a single transition (12). The second modelled switch uses a transcriptional toggle switch (28) to store the state of the device. This transcriptional switch controls the state of an inversion switch, which in turn directs transcription towards the currently inactive repressor during the next input pulse (29). Both of these devices were designed to behave as single-input binary counting modules, but to our knowledge, neither has been successfully implemented biologically.

Other large serine integrases have previously been used for different types of switching applications. Bxb1 integrase and its RDF were used to make an inversion switch that can be switched ON or OFF using different chemical inputs to induce expression of integrase, or integrase plus its RDF (5), much like pSWITCH1 with pBAD-INT and pTET[INT+RDF] in this work. Multiple integrases, without their RDFs, have been used to make state machines that irreversibly switch on gene expression in response to specific combinations of input signals in any order (4,6,7), or in specific temporal orders (8). In another work (30), the RDF of Bxb1 integrase was placed under the control of the inversion switch much like in our pSWITCH2. The negative feedback, resulting from expression of RDF as soon as the PB→LR transition starts, was shown to reduce the cell-to-cell variability of switching.

The successful operation of our single-input switch required a delay circuit, which was implemented using the tetracycline repressor, TetR. Our modelling showed how this delay circuit prevented RDF from being expressed from DNA in the LR state immediately after switching, thus allowing full PB→LR conversion. In addition, TetR expression from DNA in the PB state repressed the expression of RDF from any remaining DNA in the LR state, allowing the repeated operation of the switch over multiple cycles, despite the <100% conversion of LR→PB.

The response and recovery times of our latch will be important for its use in a binary counter. We determined the kinetics of recombination *in vivo* and showed that expression of the integrase and recombination occurred within ∼30 min (Supplementary Figure S3). In the pulsed time course experiments (e.g. Supplementary Figure S4), recombination presumably continues after the arabinose signal has been removed, but the kinetics appears to be similar. Our experiments show that pSWITCH3 works well with input pulses ranging from 10 min to 2 h, although the LR→PB transition starts to reverse during longer pulses (Supplementary Figure S9C). Our modelling suggests that low integrase expression rates should increase the range of working pulse lengths at the expense of reducing the response rate (Supplementary Figure 10D). After the end of an input signal, the levels of integrase, TetR and RDF take time to reach a steady state. This might limit how close two input signals can be before they start to blend together into a single signal. Our modelling indicated that pSWITCH3 should work well with pulses separated by >2 h, but with shorter separations the PB and LR states start to become less distinct (Supplementary Figure S14).

Our previous *in vitro* results demonstrated that stoichiometric amounts of RDF are required to activate integrase for LR→PB recombination (16). The same phenomenon was observed here *in vivo*; higher expression of integrase led to a requirement for higher RDF expression levels for efficient LR→PB recombination (Supplementary Figure S1). The stoichiometric requirement for RDF was incorporated into our mathematical models for recombination, and these models directed us to optimize switch operation by varying the RDF expression level. Similar tuning of RDF expression levels should be applicable to other devices that use serine integrases and their RDFs to record information in DNA.

Incomplete LR→PB recombination of pSWITCH3* produced cells with mixtures of plasmids in PB and LR states. One way to avoid this mixed state would be to place the switches on the chromosome, which is present at only one copy per cell in slow-growing cells. Our preliminary results show that SWITCH2 and SWITCH3 can function in single copy on the *Escherichia coli* chromosome, giving cells that are either ON or OFF, with no cells in an intermediate state (31). However, it is more difficult to tune expression levels of integrase, RDF and the TetR for efficient operation of these switches on the chromosome.

An electronic circuit that has two stable states and that toggles between these two states each time it receives an input is known as a ‘latch’ or ‘flip-flop’. The output of such a latch returns to its original state after it has received two consecutive input signals, and thus it functions as a divide-by-two frequency divider. Multiple electronic latches can be connected together, with the output of one latch driving the input of the next, to form an ‘asynchronous’ or ‘ripple’ binary counter. Each latch toggles from 0 to 1 and back again, each time it receives an input signal, passing its ‘overflow’ on to the next latch as it changes from 1 to 0 so that the whole circuit keeps track of the total number of input pulses as a binary number (Figure 1C).

Our φC31-based single-input toggle switch acts exactly as required for such a genetic latch, alternating between 0 and 1 at each input signal. To form a ripple counter that could count to 2*^N^* − 1, *N* latches, each built with an orthogonal integrase-RDF pair, would have to be chained together, with the output of each latch connected to the input of the next. Multiple orthogonal serine integrases and many of their RDFs are already known (6,11), so it should be possible to build and optimize further latches with the same architecture but using different integrases. We already have preliminary data on a version of SWITCH-2 using Bxb1 integrase and its RDF, showing that it works similarly to the φC31 version. Each latch requires an orthogonal repressor for its delay circuit. Members of the TetR family of repressors (32), or engineered CRISPR/Cas9 derivatives (33), provide good candidates for this purpose.

Finally, to connect the latches together to create a ripple counter, each latch would need to generate a short pulse of the next integrase in the cascade, every time it switches from LR to PB (Figure 1C). The required pulse generator could be implemented using a feed-forward loop (34,35), as illustrated in Supplementary Figure S15. In the PB state, the constitutive promoter in the invertible DNA segment transcribes the repressor required for the delay circuit and also a transcriptional activator that turns on transcription of the next integrase. This activator also turns on transcription of a slower acting repressor that shuts down expression of the integrase. After each LR→PB transition, transcription of the next integrase is first switched on by the activator and then switched off by the repressor. Pulse generators using this feed-forward mechanism have been successfully implemented using the LuxR transcriptional activator and the cI transcriptional repressor (36), or with RNA-based small transcription-activating RNA (STAR) activators and a CRISPRi-based transcriptional repressor (37).

In summary, we have devised and implemented a single-input, genetic, binary latch that operates at high efficiency in living cells. This design could serve as a blueprint for a reusable module that could be used in digital counters, to count and record large numbers of events. Such a recombinase-based digital device would have many possible applications in biotechnology.

## DATA AVAILABILITY

MATLAB code for all models is available at https://github.com/alex297/model-of-binary-counter-based-on-recombination-withserine- integrase.

## Supplementary Material

Supplementary Data

## Notes

Present address: Jia Zhao, Novo Nordisk (China) Pharmaceuticals Co., Ltd., Lei Shing Hong Center, Guangshunnan Avenue, Beijing 100102, China.

Present address: Alexandra Pokhilko, MRC Weatherall Institute of Molecular Medicine, John Radcliffe Hospital, University of Oxford, Headington, Oxford OX3 9DS.
