# Supplementary material for: A single-input binary counting module based on serine integrase site-specific recombination
Source: Nucleic Acids Res. 2019 Apr 8;47(9):4896–909. doi: 10.1093/nar/gkz245 (PMC6511857; doi:10.1093/nar/gkz245)
Supplement: Supplementary Data [file gkz245_supplemental_files.zip › Supplementary Data.pdf]

A single-input binary counting module based on serine integrase site-specific recombination

Supplementary Data

Jia Zhao[1], Alexandra Pokhilko[1], Oliver Ebenhöh[3,4], Susan J. Rosser*[2], and Sean D. Colloms*[1].

[1]Institute of Molecular, Cell and Systems Biology, University of Glasgow, Bower Building, Glasgow G12 8QQ, Scotland.

[2]SynthSys - Synthetic and Systems Biology, School of Biological Sciences, University of Edinburgh, CH Waddington Building, The King's Buildings, Mayfield Road, Edinburgh EH9 3JD, Scotland.

[3] Cluster of Excellence on Plant Sciences (CEPLAS), Heinrich-Heine-University, Universitätsstraße 1, D-40225 Düsseldorf, Germany

[4] Institute of Quantitative and Theoretical Biology, Heinrich-Heine-University Düsseldorf, Universitätsstraße 1, D-40225 Düsseldorf, Germany

* To whom correspondence should be addressed.

Susan Rosser Tel. +44 131 650 50 86

Email: Susan.Rosser@ed.ac.uk

Sean Colloms Tel. +44 141 330 6236; Fax: +44 141 330 4878;

E-mail: Sean.Colloms@glasgow.ac.uk

Table of Contents

Supplementary Figures

Figure S1	Expression levels of integrase and RDF	page 3
Figure S2	Intermolecular recombination produces multimers	page 5
Figure S3	Time-course of PB→LR recombination	page 6
Figure S4	Pulsed pSWITCH1 time-course	page 7
Figure S5	Pulsed pSWITCH2 time-course (+TetR)	page 8
Figure S6	Optimisation of pSWITCH2	page 9
Figure S7	Pulsed pSWITCH2* time-course (no TetR)	page 11
Figure S8	Scheme for recombination model	page 12
Figure S9	Pulsed pSWITCH3 time-course	page 13
Figure S10	Modelled efficiency of pSWITCH3 cycling	page 14
Figure S11	Modelled long-term kinetics of pSWITCH3	page 15
Figure S12	Optimisation of pSWITCH3	page 16
Figure S13	Modelled plasmid segregation	page 17
Figure S14	Modelling different intervals between pulses	page 19
Figure S15	Design for a pulse generator to connect latches	page 20

Modelling

Deterministic model	page 21
Kinetics of the counter	page 25
Stochastic modelling of plasmid segregation	page 26
Table S1 Parameter values of the model	page 28

Plasmid Sequences

Table S2 Sequences of plasmids available online page 29

References page 30

Supplementary figures

A Plasmid	RBS start	RBS strength
pBAD-INT	GAGCTCAGGAGTAACATATG	227
pBAD-INT-GTG	GAGCTCAGGAGTAACATGTG	144
pBAD-INT-126	GAGCTCCAGCTCGAGATGATTAACATATG	126
pBAD-INT-106	GAGCTCCAGCTCGAGATTATTAACATATG	106
pBAD-INT-56	GAGCTCCAGCTCGAGCTTCTTAACATATG	56

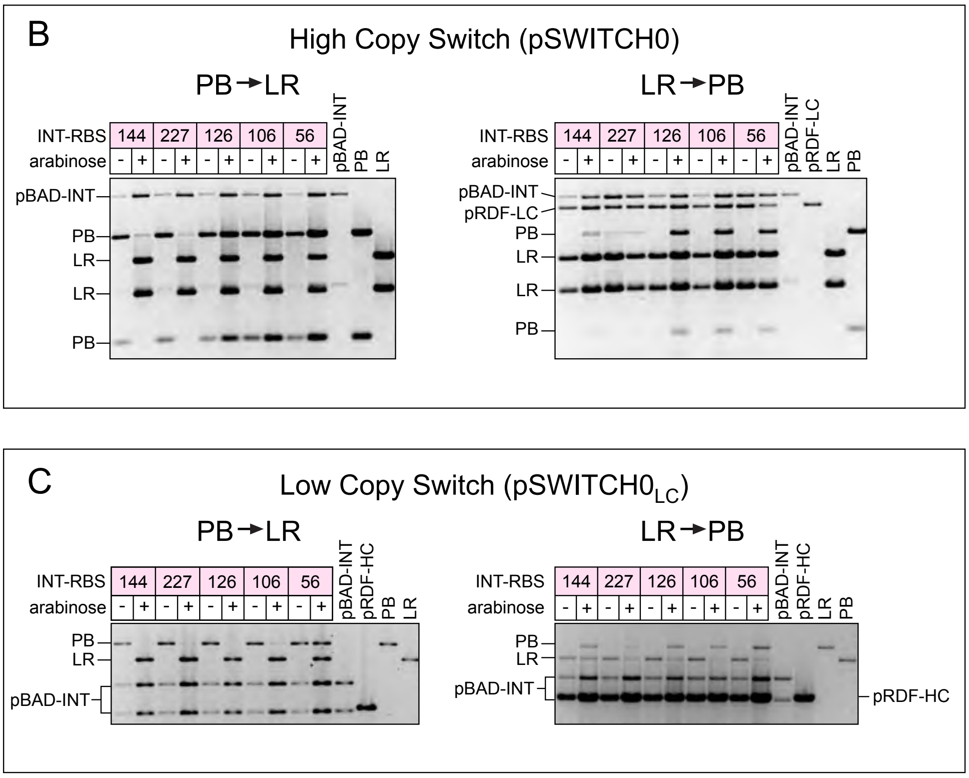

Figure S1. Expression levels of integrase and RDF, and copy number of plasmids carrying DNA inversion switches, affect switching efficiency. (A) The expression level of integrase from pBAD-INT was reduced by changing the ATG start codon to GTG, or by changing the ribosome binding site between SacI (yellow) and NdeI (green) sites as shown. Altered ribosome binding sites contain an XhoI site (blue) for diagnostic purposes. Relative translation initiation rates (RBS strength) were predicted using the RBS calculator software of Salis *et al.* (1). **(B)** Recombination from PB→LR on a high copy number (pMB1 origin) switch plasmid (pSWITCH0$_{HC}$-PB) was assayed with different strengths of integrase expression (left). Similarly, LR→PB recombination was analysed starting from pSWITCH0$_{HC}$-LR, with ϕC31 RDF expressed from a pSC101-based low copy number plasmid (pRDF-LC) and integrase expressed from pBAD-INT and derivatives as indicated (right). *E. coli* DS941 carrying the switch plasmid was transformed with integrase and RDF expression plasmids and grown overnight in the presence of 0.2% glucose (-) or 0.2% arabinose (+)

as indicated above the gels. Plasmid DNA was isolated, digested with NheI and XmnI and analysed by agarose gel electrophoresis. Fragments corresponding to PB and LR states are indicated. PB→LR recombination was most efficient at the higher levels of integrase expression (pBAD-INT and pBAD-INT-GTG). LR→PB recombination was most efficient at intermediate levels of integrase expression (pBAD-INT-126 and pBAD-INT-106). At high integrase expression levels, it appears that the amount of RDF present in the cells is not sufficient to activate integrase for LR→PB recombination, while at lowest integrase expression level (pBAD-INT-56) integrase appears to be limiting. **(C)** Recombination from PB→LR and LR→PB on a low copy number (pSC101 origin) switch plasmid (pSWITCH0-LC) was assayed as in (B) except that DNA was digested with NheI and AlwNI prior to agarose gel electrophoresis. For LR→PB recombination, a high copy number RDF expression plasmid (pRDF-HC) was used. PB→LR recombination was efficient with all except the weakest integrase expression plasmid (pBAD-INT-56). LR→PB recombination took place at all levels of integrase expression but was most efficient at the lower integrase levels (pBAD-INT-106 and pBAD-INT-56). Glucose was included in overnight cultures as a "catabolite repressor" to reduce leaky expression of integrase from the arabinose-regulated P_{BAD} promoter. As well as repressing transcription from P_{BAD}, glucose leads to lower levels of plasmid recovery from overnight cultures of *E. coli*. This is most noticeable for the p15a-origin pBAD-INT and the pMB1-origin pSWITCH0 and pRDF-HC plasmids in both (B) and (C). Metabolism of glucose by *E. coli* at high growth rates leads to the accumulation of acetate and this is thought to reduce plasmid copy number as well as the overall yield of biomass (2,3).

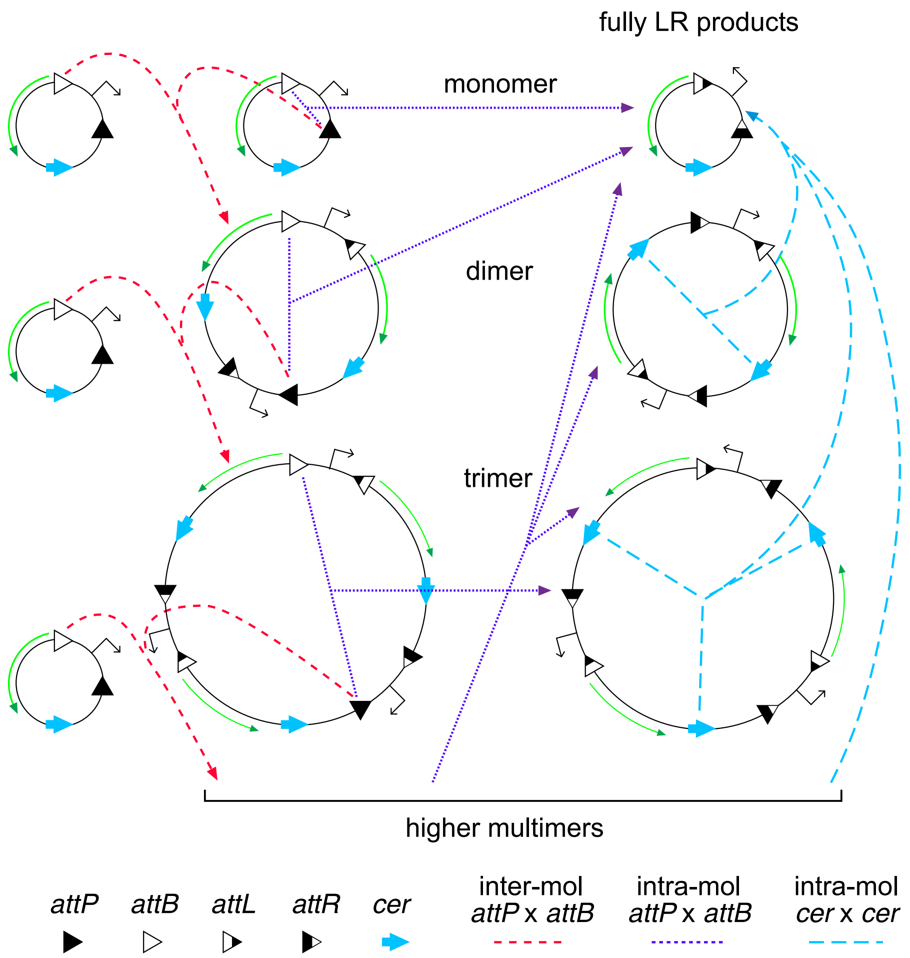

Figure S2. Intermolecular integrase-mediated recombination produces multimers that can be resolved to monomers by Xer site-specific recombination at *cer*. Recombination between *attP* and *attB* sites on the same inversion switch plasmid (intramolecular, purple dotted lines) produces monomeric LR product. Intermolecular recombination between *attP* on one monomer and *attB* on another monomer (intermolecular, red dashed lines) produces dimers containing *attL*, *attR* and un-recombined *attP* and *attB* sites. Further intermolecular PB recombination produces trimers and higher multimers, which always contain one unrecombined *attP* and one un-recombined *attB* site. These unrecombined *attP* and *attB* sites can recombine intramolecularly to give products that are fully in the LR state. This combination of inter- and intramolecular integrase-mediated recombination continues until all *attP* and *attB* sites have been converted to *attL* and *attR* and produces all multimers (dimers, trimers, tetramers etc) of the fully LR product. The products of restriction digestion of these multimers will be indistinguishable from those of the LR monomer. Site-specific recombination at *cer* is mediated by the chromosomally encoded *E. coli* Xer recombination system and occurs exclusively between directly repeated (head-to-tail) *cer* sites (light blue arrow heads) on the same circular DNA molecule (4-7). Fully LR multimers contain directly repeated *cer* sites so that Xer recombination will produce the LR monomeric product (blue dashed lines).

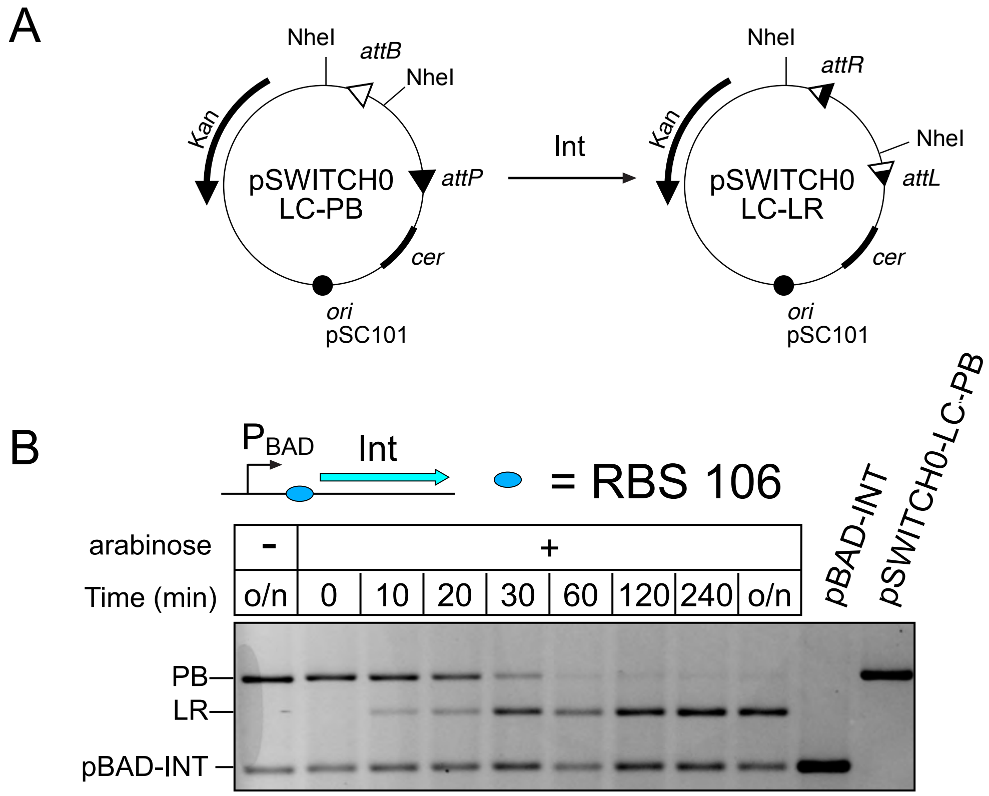

Figure S3. Time-course of PB→LR recombination. (A) Restriction maps of PB and LR forms of pSWITCH0-LC plasmid used in the time course assay. **(B)** Time-course of PB→LR recombination on pSWITCH0-LC. *E. coli* DS941 cells containing pSWITCH0LC-PB and the integrase expression plasmid pBAD-INT-106 were cultured overnight in LB broth with 0.2% glucose to repress integrase expression. The overnight culture was diluted 40-fold in fresh LB broth without glucose and incubated for 90 minutes with shaking at 37 °C to reach mid-exponential phase before adding arabinose to 0.2%. At the indicated time after arabinose addition, cells were harvested by centrifugation and frozen in liquid nitrogen. Plasmid DNA was extracted from equivalent amounts of cell pellet at all time points, digested with XmnI and NheI and analysed by agarose gel electrophoresis. Lane 1 (“-” arabinose “o/n”) shows DNA isolated from the original overnight culture grown in the presence of glucose. Lane 9 (“+” arabinose “o/n”) shows DNA isolated from cells grown overnight for 18 hours after addition of arabinose. The final two lanes show the integrase expression plasmid (pBAD-INT-106) and pSWITCH0-LC in the PB state (pPB). pSWITCH0-LC yields NheI fragments of 4562 bp and 92 bp in the PB state, and 3848 bp and 806 bp in the LR state. XmnI does not cleave pSWITCH0-LC but was used to prevent bands from pBAD-INT-106 co-migrating with those from pSWITCH0-LC. XmnI - NheI cleavage of pBAD-INT-106 yields fragment of 2984 bp, 2233 bp, 1504 bp, 411 bp, and 52 bp. Fragments smaller than 2500 bp have been cropped from the gel picture. In this and all other figures, *att* sites are represented as triangles pointing 5’ to 3’ in the direction of the “top” strand containing the sequence 5’-TT-3’ as central dinucleotide.

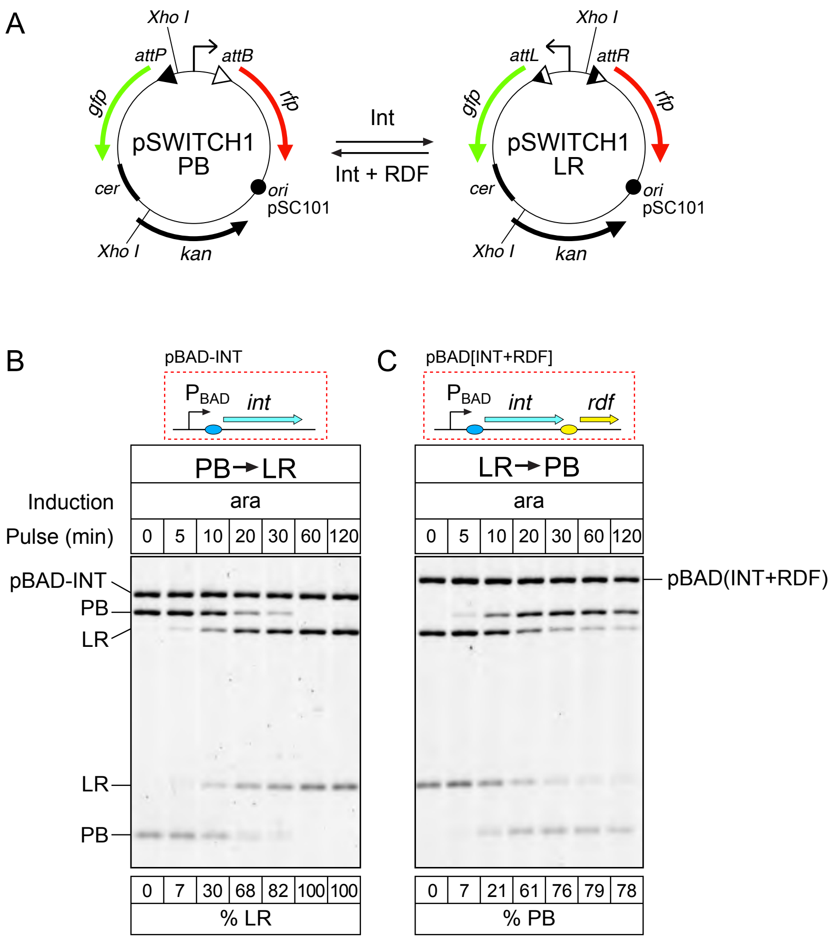

Figure S4 Time-course of pSWITCH1 PB→LR and LR→PB recombination using pulsed expression of integrase. (A) Restriction maps of PB and LR forms of pSWITCH1 plasmid used in the pulsed arabinose time course assay. **(B)** Time-course of PB→LR recombination on pSWITCH1. *E. coli* DS941 cells harbouring pSWITCH1-PB and the integrase expression plasmid pBAD-INT were cultured overnight in LB broth with 0.2% glucose to repress integrase expression. The overnight culture was diluted 40-fold in fresh LB broth without glucose and incubated for 90 minutes with shaking at 37 °C. Arabinose was added to 0.2% and at the indicated time after arabinose addition, samples were withdrawn and diluted 1000-fold into fresh LB containing 0.2% glucose. Plasmid DNA was purified after overnight growth, digested with XhoI, and run on an agarose gel. Bands from the substrate (PB), product (LR) and integrase expression plasmids are indicated. The fraction of DNA in the LR state was quantitated and is indicated below each lane. **(C)** Time-course of LR→PB recombination on pSWITCH1, carried out as in B except that DS941 cells started with pSWITCH1-LR and pBAD[INT+RDF]. The fraction of DNA in the PB state is indicated below each lane.

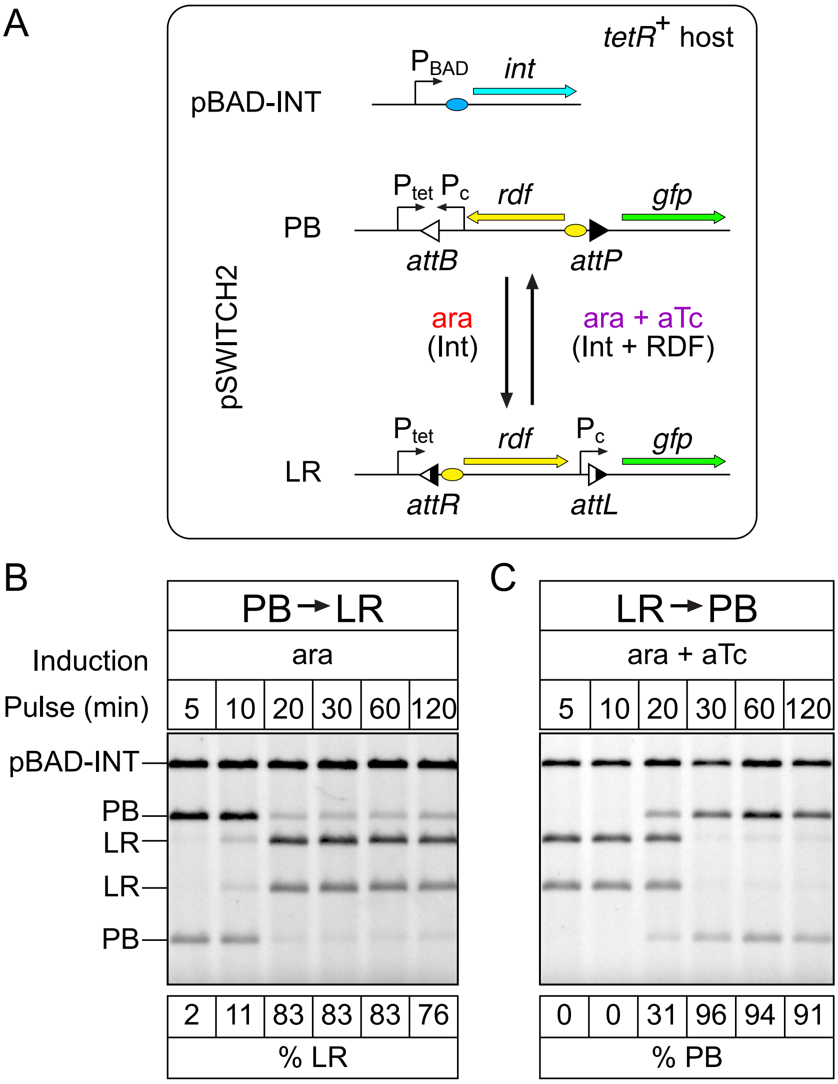

Figure S5. Pulsed induction time-course of pSWITCH2 PB→LR and LR→PB recombination in the presence of TetR. (A) The non-optimised pSWITCH2, with RDF expressed from the aTc-inducible promoter $P_{LtetO-1}$ (P_{tet}) only in the LR state, was introduced into a *tetR*$^{+}$ host (*E. coli* DS941 Z1) together with the arabinose-inducible integrase expression plasmid pBAD-INT. **(B)** Pulsed time-course of PB→LR recombination in DS941 Z1 containing pSWITCH2-PB and pBAD-INT. Arabinose (0.2 %) was added to induce integrase expression at time 0. Samples were removed at the indicated time after induction and stopped by 1000-fold dilution into LB broth containing 0.2% glucose. After overnight growth, plasmid DNA was isolated, digested with SpeI and run on an agarose gel. The fraction of DNA in the LR state was quantitated and is indicated below each lane. **(C)** Pulsed time-course of LR→PB recombination in DS941 Z1 containing pSWITCH2-LR and pBAD-INT. Arabinose (0.2%) and anhydrotetracycline (100 ng/ml) were added to induce integrase and RDF. Plasmid DNA was isolated and digested with SpeI prior to agarose gel electrophoresis. The fraction of DNA in the PB state is indicated below each lane.

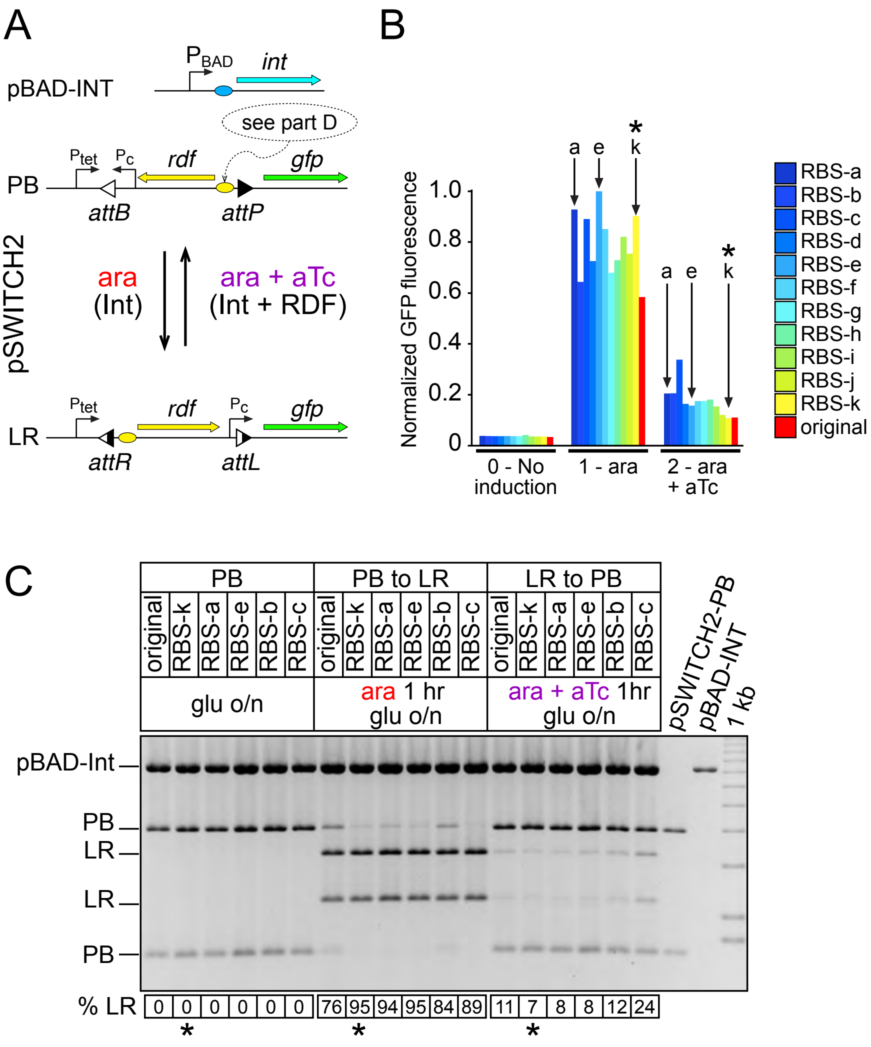

D

	RBS sequence	predicted translation initiation rate (au)
original	AAGAAGGAGATATAAAT**ATG**	19,612
library	AAGNRRGNGRTATAAAT**ATG**	
RBS-k*	AAGTAAGTGGTATAAAT**ATG**	1,106
RBS-a	AAGAAAGTGGTATAAAT**ATG**	644
RBS-e	AAGGGAGTGGTATAAAT**ATG**	3,989
RBS-b	AAGCGGGAGGTATAAAT**ATG**	23,586
RBS-c	AAGGGGGTGGTATAAAT**ATG**	34,733

Figure S6. Optimisation of pSWITCH2 PB→LR and LR→PB recombination in the presence of TetR. (A) Diagrammatic representation of the state-based switch in pSWITCH2. In a *tetR*$^+$ host, pSWITCH2 should recombine from PB→LR in the presence of arabinose (switching GFP on), and from LR→PB in the presence of arabinose plus aTc (switching GFP off). The ribosome binding site (RBS) for the *rdf* gene was randomised to AAGNRRGNGRTATAAAT**ATG** (N = A, C, G or T; R = G or A) giving 128 possible variants. **(B)** The mutant library was generated in the LR (GFP on) state, introduced into DS941 Z1 containing pBAD-INT and induced with a one-hour pulse of arabinose plus aTc. Cells were diluted onto selective plates and colonies containing pSWITCH2 that had successfully changed to the PB (GFP off) state were selected as non-fluorescent after scanning in a

fluorescence imager. 11 randomly chosen colonies from this initial screen (RBS-a to k) were then tested for their response to 1-hour pulsed induction with arabinose (1-ara), followed the next day by one-hour pulsed induction with arabinose and aTc (2-ara+aTc). The bar chart shows the GFP fluorescence of 100 μl samples of liquid cultures at each stage, measured in 96-well plates. The original pSWITCH2 (original) was used as a control. Promising candidates (a, e and k) had high GFP fluorescence after the arabinose pulse and low fluorescence after the arabinose plus aTc pulse. **(C)** Comparison of PB→LR and LR→PB recombination on the original pSWITCH2 (original) and variants with mutant ribosome binding sites (RBS-a, RBS-b, RBS-c, RBS-e and RBS-k). DS941 Z1 cells containing pSWITCH2 and its RBS mutant derivates, as well as pBAD-INT, were grown overnight in the presence of glucose (glu o/n), induced with a 1-hour pulse of arabinose and allowed to grow to stationary phase in broth containing 0.2% glucose (ara 1 hr glu o/n). These cells were then diluted in fresh media and induced with a 1-hour pulse of arabinose plus aTc (ara + aTc 1 hr glu o/n). Plasmid DNA was isolated, digested with SpeI and subjected to agarose gel electrophoresis. The percentage of DNA in the LR state is shown below each lane. **(D)** Sequences of ribosome binding sites for the original pSWITCH2 and the five variants shown in (C). Relative translation initiation rates (RBS strength) were predicted using the RBS calculator software of Salis (1). Weaker RBS sequences showed improved switching. The RBS-k mutant was chosen as the optimized pSWITCH2* for subsequent experiments.

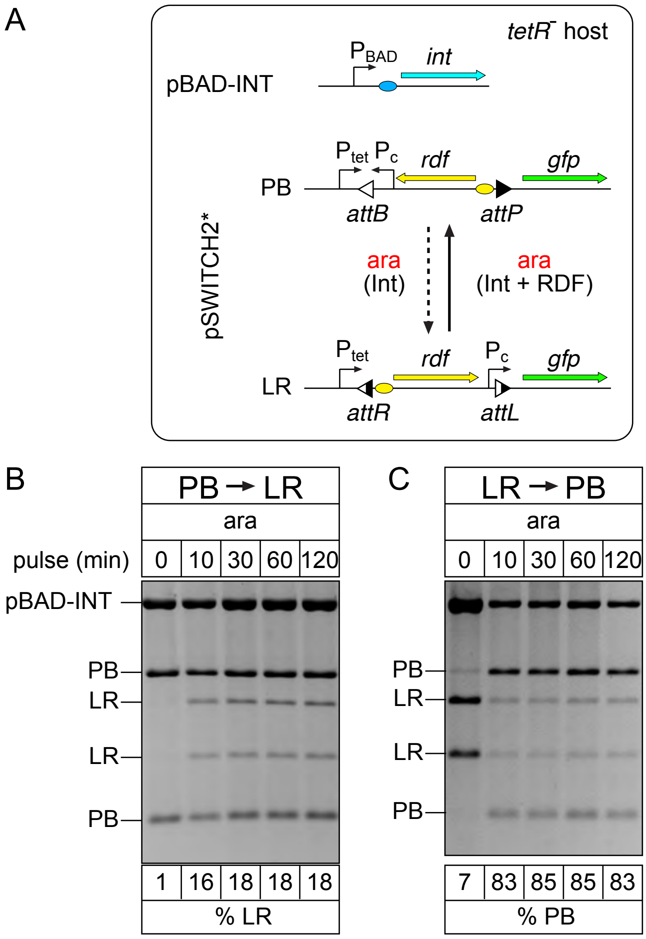

Figure S7. Pulsed induction time-course of pSWITCH2* PB→LR and LR→PB recombination in the absence of TetR. (A) The optimized pSWITCH2* was introduced into a *tetR⁻ E. coli* host (DS941) together with the arabinose-inducible integrase expression plasmid pBAD-INT. $P_{LtetO\text{-}1}$ (P_{tet}) is constitutively active in DS941, so the RDF will be expressed whenever the switch is in the LR state. According to the original design, addition of arabinose in the PB state will lead to expression of integrase and conversion to LR, switching on expression of RDF. In the LR state, arabinose induction of integrase (with RDF now present) would lead to conversion back to PB. **(B)** Pulsed time-course of PB→LR recombination in DS941 containing pSWITCH2*-PB and pBAD-INT. Arabinose (0.2%) was added to induce integrase expression at time 0. Samples were removed at the indicated time after induction and stopped by 1000-fold dilution into LB broth containing 0.2% glucose. After overnight growth, plasmid DNA was isolated, digested with SpeI and run on an agarose gel. The fraction of DNA in the LR state was quantitated and is indicated below each lane. **(C)** Pulsed time-course of LR→PB recombination in DS941 containing pSWITCH2*-LR and pBAD-INT. Arabinose (0.2%) was added to induce integrase. Plasmid DNA was digested with SpeI prior to agarose gel electrophoresis. The fraction of DNA in the PB state is indicated below each lane. The switch appears to reach the same steady state (~80% PB) starting from either PB or LR, presumably due to rapid expression of the RDF as soon as a small amount of LR is present.

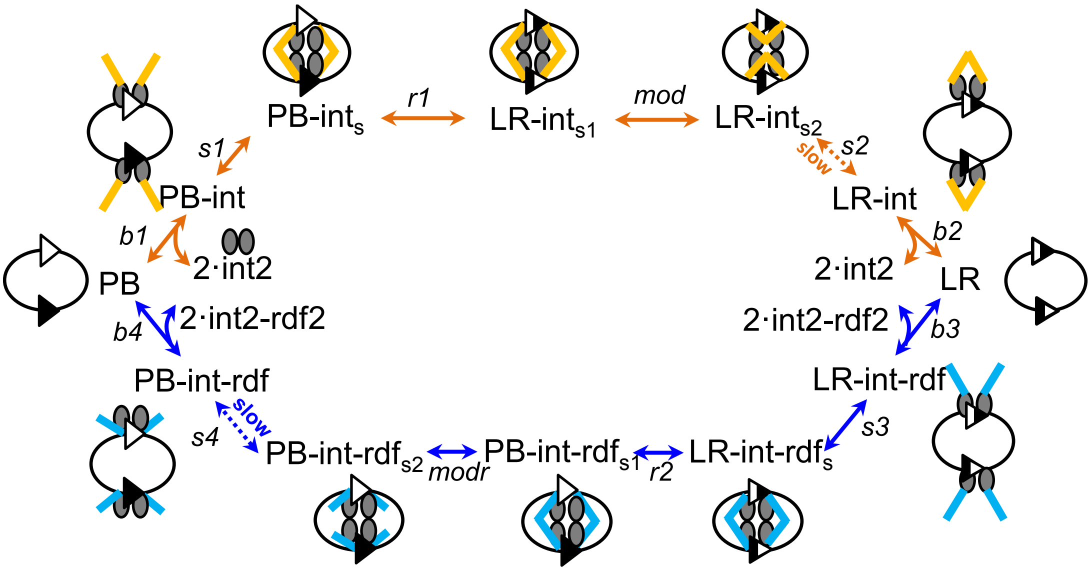

Figure S8. Scheme used to model recombination reactions. In the absence of RDF, reactions start from binding of the PB substrate to two integrase dimers (int2), leading to the formation of tetrameric non-synaptic (PB-int) and synaptic (PB-int$_s$) complexes. PB-int$_s$ undergoes recombination resulting in the LR-int$_{s1}$ complex, which changes its conformation to produce LR-int$_{s2}$. LR-int$_{s2}$ then can slowly desynapse and release free integrase dimers. In presence of RDF, reactions start from binding of the LR substrate to two heterotetrameric complexes of Int and RDF (int2-rdf2), forming non-synaptic (LR-int-rdf) and synaptic (LR-int-rdf$_s$) complexes. LR-int-rdf$_s$ undergoes recombination resulting in PB-int-rdf$_{s1}$ complex, which changes its conformation to produce PB-int-rdf$_{s2}$. PB-int-rdf$_{s2}$ then can slowly desynapse and release free PB product. Orange and blue coloured arrows correspond to integrase reactions without or with RDF respectively. Figure redrawn from Pokhilko *et al*. (8).

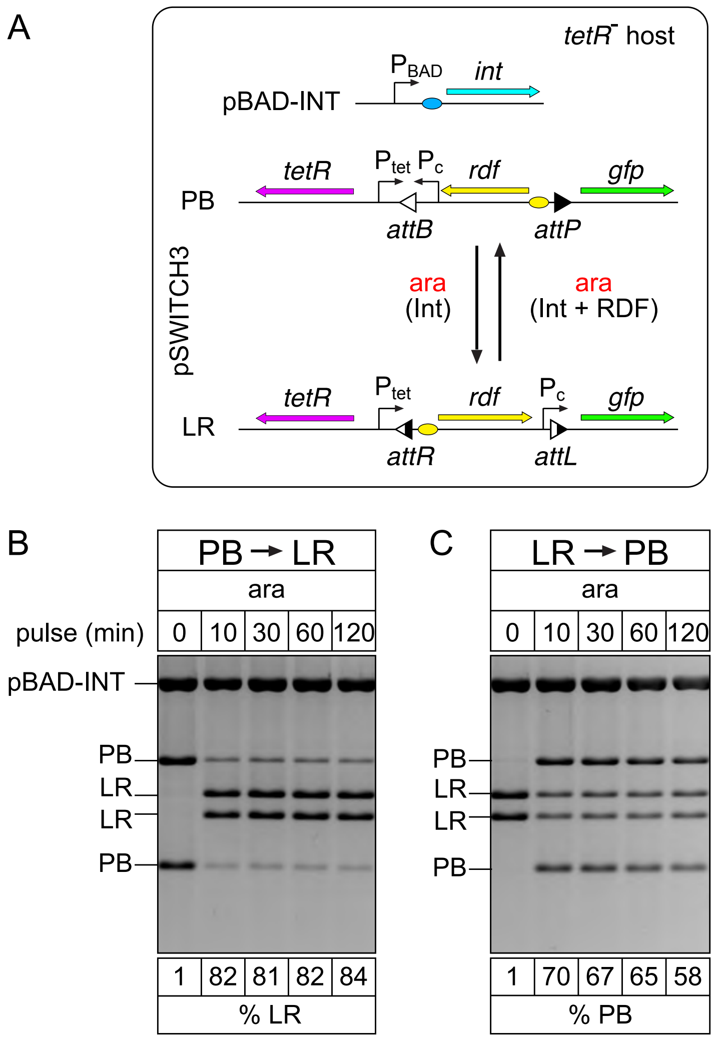

Figure S9. Pulsed induction time course of pSWITCH3 PB→LR and LR→PB recombination. **(A)** The non-optimised binary counter module (pSWITCH3) was created by introducing the *tetR* gene to the left of the invertible segment in pSWITCH2. Plasmid pSWITCH3 was introduced into a *tetR*⁻ host (*E. coli* DS941) together with the arabinose-inducible integrase expression plasmid pBAD-INT. **(B)** Starting from pSWITCH3 in the PB state or **(C)** in the LR state, cells were cultured in LB broth with 0.2% glucose overnight. The overnight culture was diluted 40-fold in fresh LB broth without glucose and incubated for 90 minutes shaking at 37 °C to mid-exponential phase. Integrase expression was induced by adding 0.2% arabinose. At the indicated times, samples of cells were removed from the culture and diluted 1000-fold into LB broth containing 0.2% glucose to stop further expression of integrase. After overnight growth, plasmid DNA was purified and digested with SpeI prior to agarose gel electrophoresis. Diagnostic fragments for PB and LR states are indicated to the left of the gels, and the percentage of plasmid DNA in the LR (B) or PB (C) state is indicated below each lane.

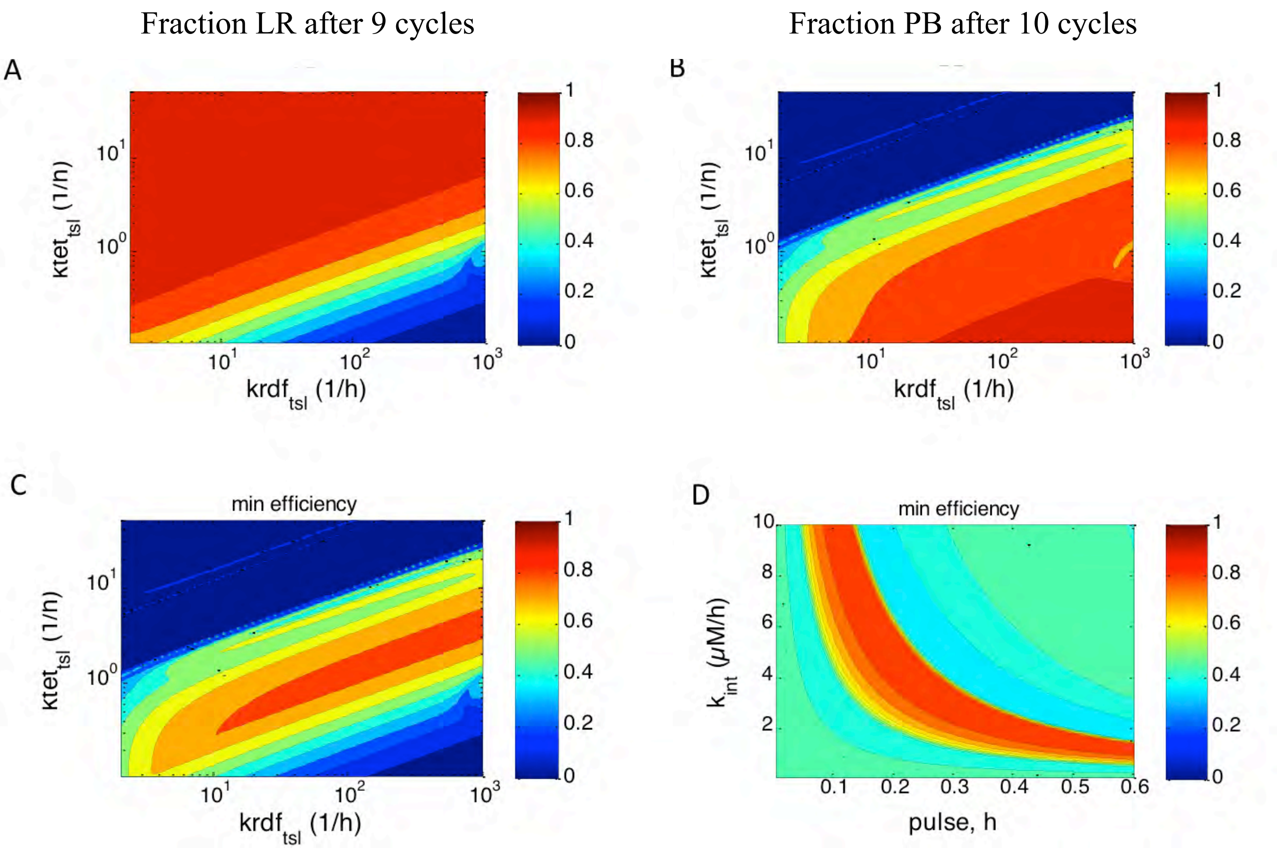

Figure S10. Simulated efficiencies of pSWITCH3 cycling with different expression levels for RDF and TetR. The inversion of pSWITCH3 (with expression of TetR regulated by the switch, as in Main Text Figure 5D or Supplementary Figure S9A) was modelled over ten 24-hour cycles, each with a single 12-minute arabinose pulse. The switch is started in the PB state, and if switching is efficient, it will alternate between PB and LR states at each cycle. For each value of the rate constants governing the expression of RDF and TetR (k_{rdf_tsl} and k_{tetR_tsl}), **(A)** Shows the maximum fraction of DNA molecules in the LR state after the ninth arabinose pulse, while **(B)** shows the maximum fraction of switch molecules in the PB state after the tenth arabinose pulse. If efficient switching is occurring after each arabinose pulse, the switch should be in the LR state after 9 pulses and in the PB state after 10 pulses. Therefore, values of RDF and TetR expression that give efficient cycling between PB and LR states are coloured red in both (A) and (B). Areas that are red in (A) and blue in (B) correspond to switches that get trapped in the LR state after multiple pulses, while those that are blue in (A) and red in (B) correspond to switches that get trapped in the PB state. **(C)** Overall cycling efficiency of the switch in both directions (PB→LR and LR→PB) as a function of translation rates of RDF and TetR, calculated as the minimum of the plots shown in A and B. The model kinetics shown in Main Text Figure 5 and Supplementary Figure S11 correspond to $k_{rdf\_tsl} = 4\ h^{-1}$ and $k_{tetR\_tsl} = 0.3\ h^{-1}$. **(D)** Dependence of the overall cycling efficiency of the switch (as in C) on the duration of arabinose pulse (ara_{on} - ara_{off}) and the rate constant k_{int} of the production of Int protein, for the optimized rates of RDF and TetR translation: $k_{rdf\_tsl} = 800\ h^{-1}$, $k_{tetR\_tsl} = 3.4\ h^{-1}$.

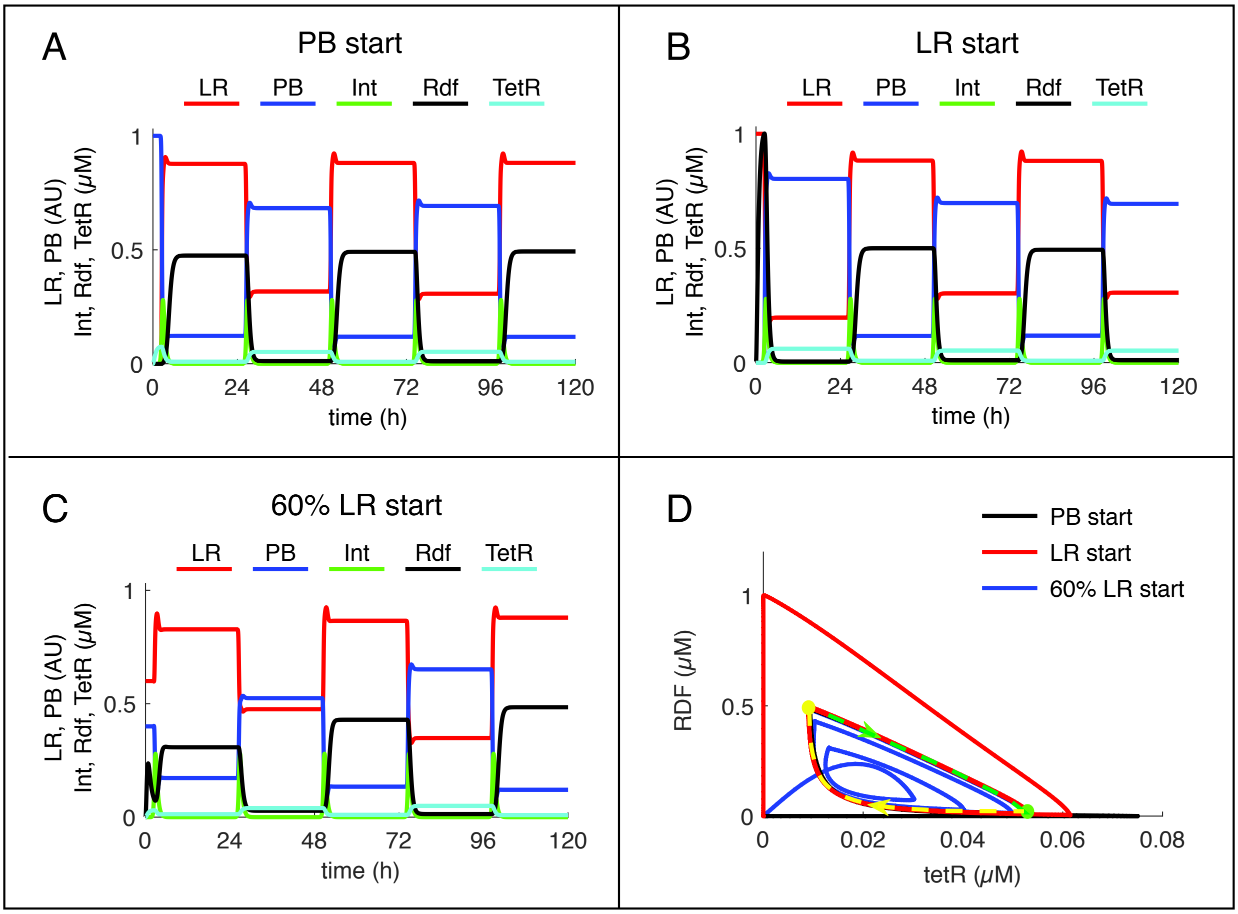

Figure S11. Modelling of the long-term kinetics of pSWITCH3. A first pulse of arabinose was given at 2 h; subsequent pulses were given at 24 h intervals. All pulses were 12 minutes in duration. **(A-C)** The kinetics of the relative amounts of LR (red), PB (blue) and concentrations of Int (green), RDF (black) and TetR (cyan) for different initial starting conditions: **(A)** starting from 100% PB, **(B)** starting from 100% LR and **(C)** starting from 60% LR and 40% PB. Amounts of PB and LR are shown in normalised units (AU), protein concentrations are in μM. **(D)** Phase diagram showing changes in TetR and RDF concentrations over 10 cycles (24 h each). Trajectories, which start from 0%, 100% and 60% of LR (and zero TetR and RDF) are shown by black, red and blue lines respectively. Independently of initial conditions, after several cycles all trajectories end up on a cyclic trajectory, fluctuating between 2 steady states with high LR and RDF (yellow dot) and high PB and TetR (green dot). The LR→PB and PB→LR transitions are shown by green and yellow dashed lines respectively.

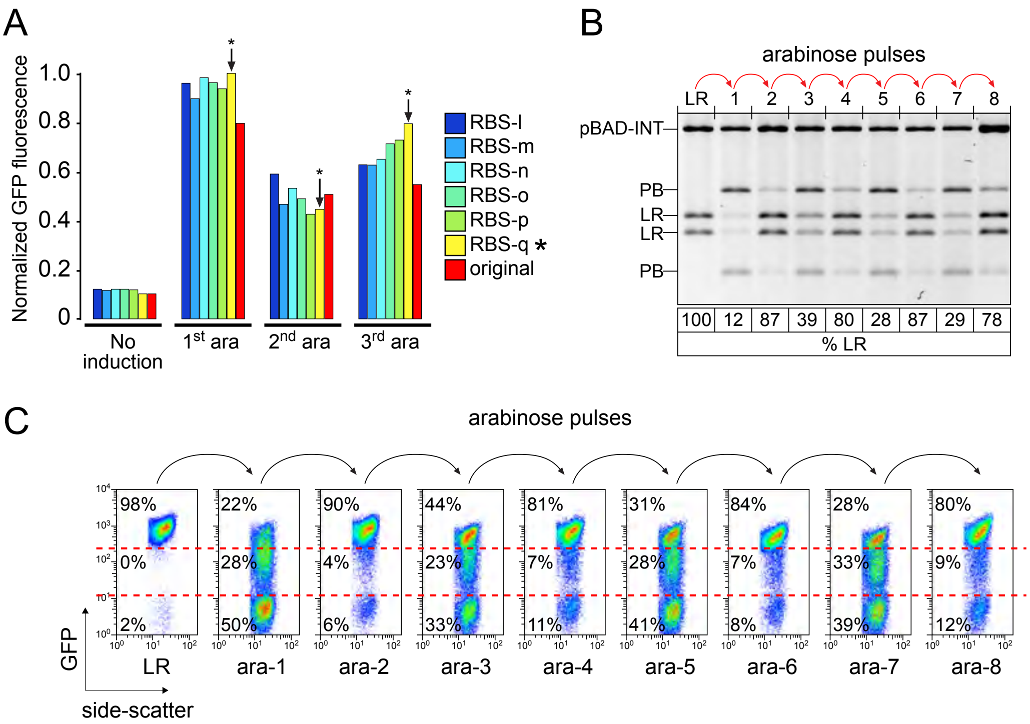

Figure S12. Optimisation of the binary counting module. (A) The expression level of RDF in pSWITCH3 was optimised using a similar strategy to that used for pSWITCH2 (see Figure S6). The RDF RBS library was inserted into pSWITCH3 in the LR state and candidates that retained LR→PB switching were picked as colonies with low fluorescence after a single 1-hour arabinose pulse. The bar chart shows the behaviour of six pSWITCH3 variants isolated in this way (RBS-l, m, n, o, p, and q) over three arabinose pulses starting from the pure PB (GFP off) state. The original pSWITCH3 (original) was used as a control. The mutant version of pSWITCH3 with RBS-q (indicated with an asterisk (*)) was chosen as the optimised pSWITCH3*. Its RBS (AAGTGAGCGGTATAAAT; predicted initiation rate = 393 AU; (1)) had a lower predicted translation initiation rate than the original one (AAGAAGGAGATATAAAT; predicted initiation rate = 19,612; (1)), improving the transition from PB→LR during the 1st and 3rd arabinose pulses. **(B)** Operation of pSWITCH3* over eight cycles of pulsed integrase expression starting from the LR state. This experiment was identical to that shown in Figure 6 in the Main Text except that pSWITCH3* started in the LR state. For each cycle, exponentially growing cells were exposed to 0.2% arabinose for 15 minutes to induce integrase expression, followed by 1:1000 dilution into LB broth containing 0.2% glucose to repress further expression. After overnight growth, plasmid DNA was purified and analysed by restriction digestion and agarose gel electrophoresis. The percentage of DNA in the LR state is shown below each lane. **(C)** GFP fluorescence of approximately 30,000 cells was measured after each overnight culture by flow cytometry. Percentages of cells in low, intermediate and high fluorescence states are shown on the plots.

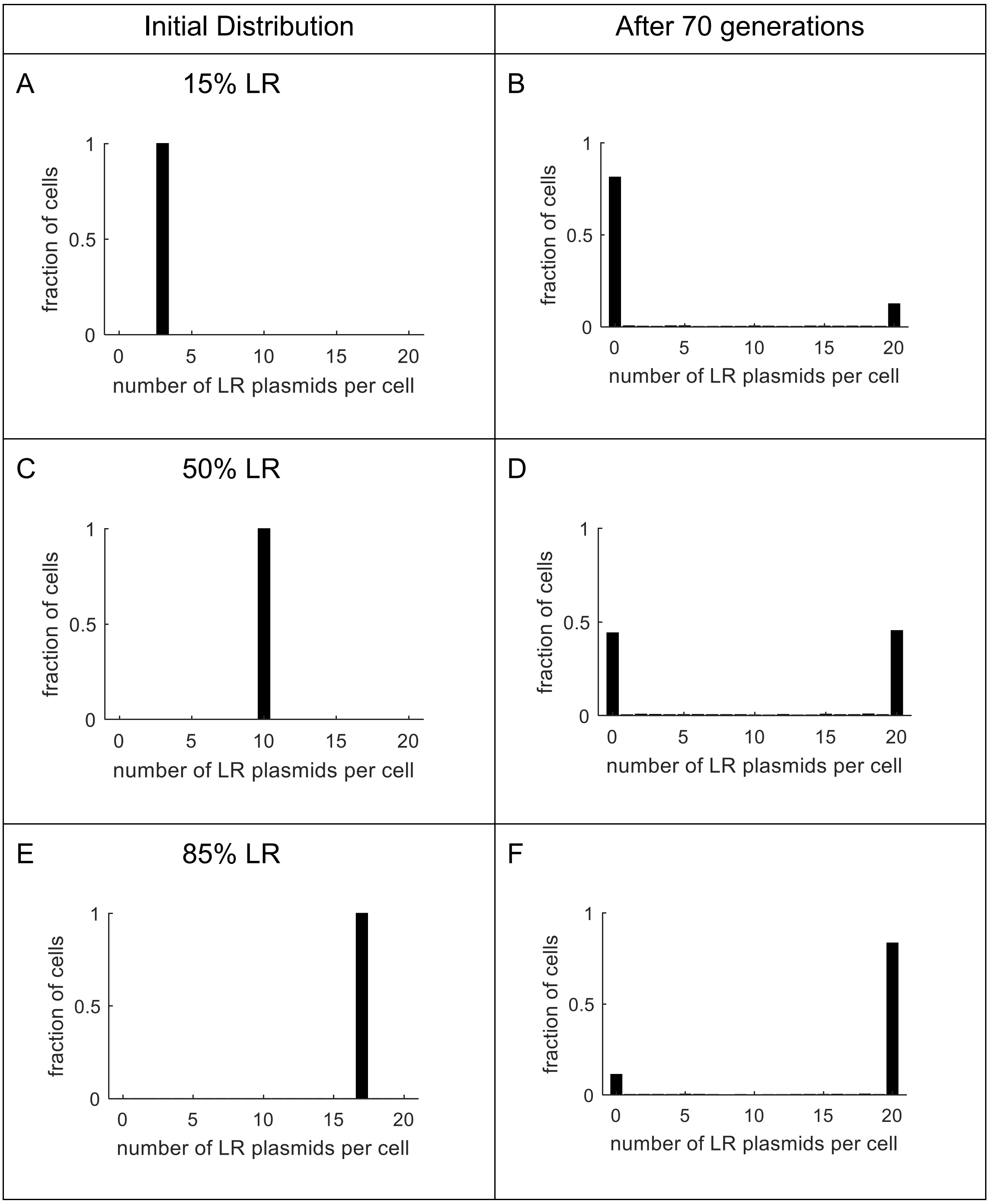

Figure S13. Modelled plasmid segregation starting from different ratios of PB:LR in all cells. Random plasmid segregation for a pSWITCH plasmid with 20 copies at cell division was modelled for 70 generations starting from different initial levels of PB and LR in all cells. The graphs on the left (A, C, E) show the initial distribution of PB and LR plasmids in cells, and the graphs on the right (B, D, F) show the predicted distribution after 70 generations of growth. Simulations were carried out as described in the legend of Figure 7 in the Main Text. (A, B) Initially all cells contained 3

plasmid copies in the LR state and 17 in the PB state. (C, D) Initially all cells contained 10 PB and 10 LR copies. (E, F) Initially all cells contained 17 LR and 3 PB plasmids. C. After 70 cell divisions, almost all cells contain only PB or only LR copies, and the proportion of these cells reflects the original abundance of the two plasmid states in the starting population.

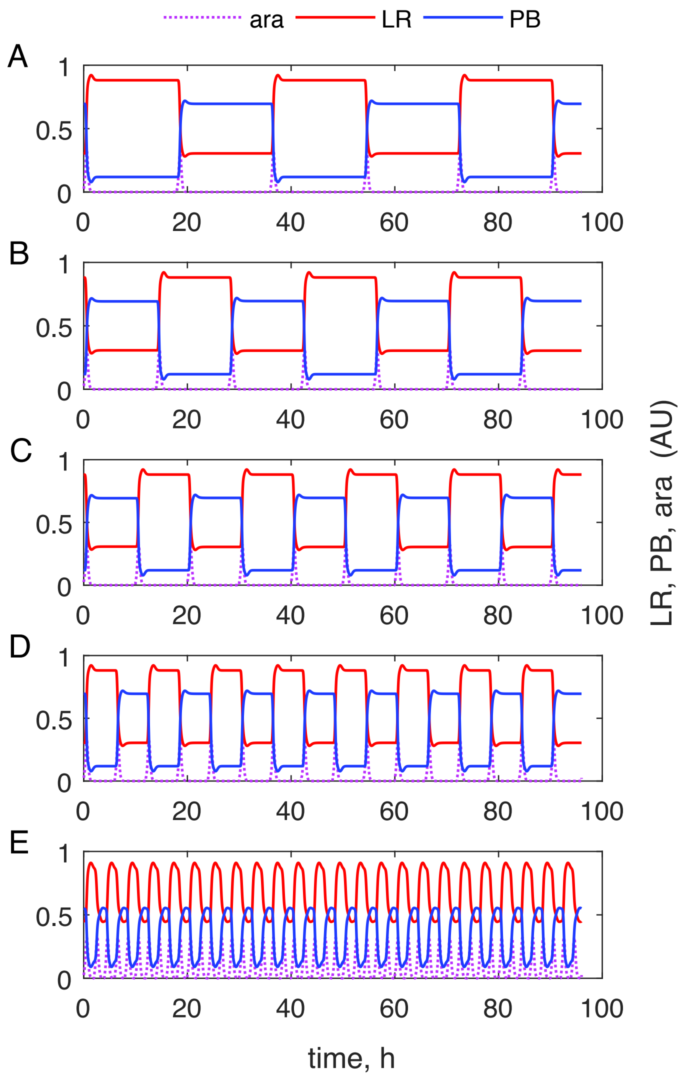

Figure S14. Modelling different time intervals between arabinose pulses. The model for pSWITCH3 was used with 12-minute arabinose pulses repeated every (A) 18 hours, (B) 14 hours, (C) 10 hours, (D) 6 hours and (E) 2 hours. The parameters used were as follows: ara_on = 0.5 h; ara_off = 0.7 h; period = 18, 14, 10, 6, or 2 h. Simulations were pre-run for 144 hours before time 0, starting from the PB state, to reach steady oscillating trajectories for each set of induction parameters.

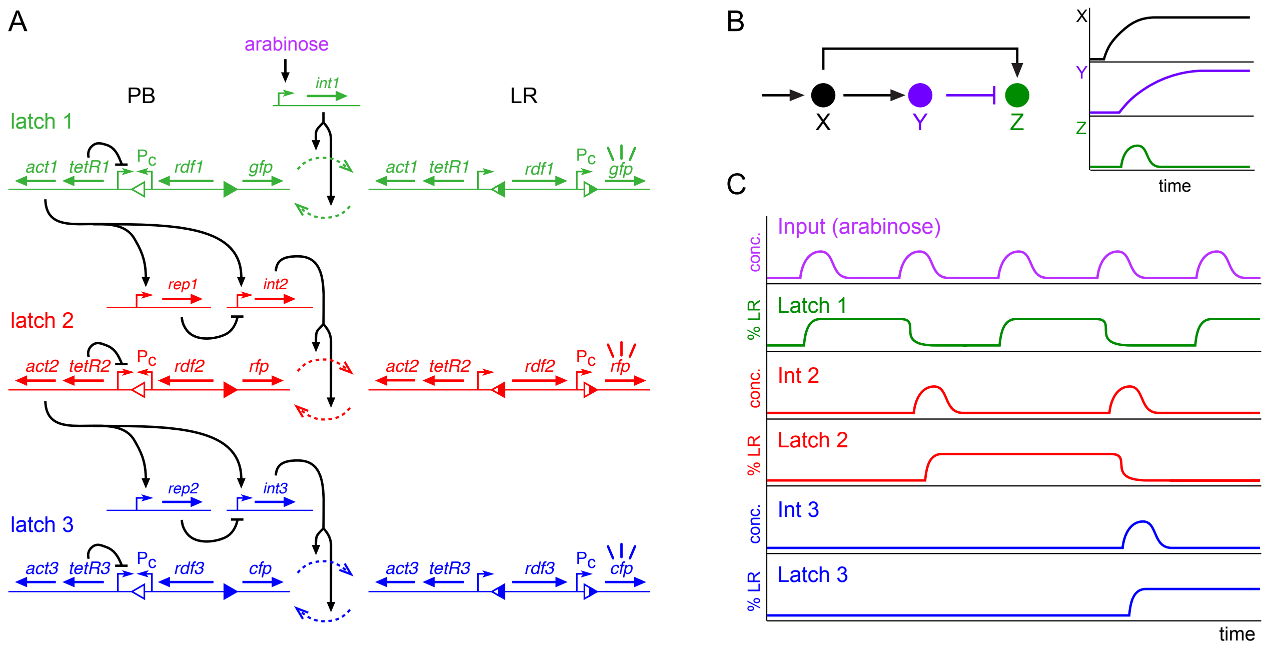

Figure S15. Linking latches together using a feed-forward loop. (A) Proposed genetic circuit to form a 3-bit binary counter. Three flip-flop latches are linked together to form a 3-bit counter. Each latch uses the same SWITCH3 architecture, but with three different orthogonal integrases (*int1*, *int2* and *int3*) and their RDFs (*rdf1*, *rdf2*, and *rdf3*) and *att* sites. Latch 1 drives expression of GFP, latch 2 drives expression of RFP and latch 3 drives expression of CFP only when in their LR states. Each latch utilises an orthogonal repressor from the TetR family (*tetR1*, *tetR2* and *tetR3*) for their delay circuit. In addition, each switch expresses a transcriptional activator (*act1*, *act2* and *act3*) only in the PB state. Each of these activators turns on transcription of the integrase for the next latch, and also a repressor protein (*rep1*, *rep2* and *rep3*) that switches off transcription of this integrase. Latch 1 is toggled between PB and LR states by an external signal (arabinose). Each activator-repressor pair (*act1* with *rep1*; *act2* with *rep2* etc.) forms a feed-forward loop to generate an expression pulse of the next integrase, toggling the state of the next latch. The three latches are coloured green, red and blue, and interactions between components are indicated by black lines. **(B)** Function of the type 1 incoherent feed-forward loop (9) to generate pulsed gene expression. The input signal switches on expression of a transcriptional activator (X; *act1*, *act2* or *act3* in the binary counter modules), which activates expression of the output (Z; the next integrase) and a repressor (Y; *rep1*, *rep2* or *rep3*). The graph shows the predicted production of X, Y and Z over time. Production of X activates production of Y and Z with a slight delay. When Y reaches some threshold, it inhibits further production of Z. **(C)** Predicted response of the 3-bit counter to 5 input pulses over time. The graphs show the expected changes in concentrations of arabinose, int2 and int3, and the states of each latch over time. Each latch acts as a divide-by-two frequency divider, and the complete circuit acts as a binary counter.

Modelling

Deterministic model

The model for recombination by ϕC31 integrase (**Int**) with or without RDF was developed in (8). Here we extend this model to include equations describing the intracellular kinetics of the counter with and without TetR (see Main Text Figure 5A,D). In particular, we added equations for the production of Int, RDF and TetR proteins. The *int* gene is located on a separate plasmid; therefore the production of Int does not depend on the DNA state (PB or LR) and Int is assumed to be induced in the presence of an arabinose pulse. The levels of RDF and TetR proteins are determined by their mRNA levels, which in turn depend on the DNA state. TetR is expressed in the PB states and RDF is expressed in the LR state; RDF expression is inhibited by TetR protein (Main Text Figure 5D). The decay of Int, RDF and TetR proteins (k_{dil}) was assumed to be determined by dilution due to cell division (assuming a doubling time of 20 minutes). Int and RDF proteins were assumed to be diluted from their DNA complexes by DNA replication ($t_{1/2}$ ~ 20 min), releasing free DNA (PB or LR). The rate constant of *rdf* and *tetR* mRNA degradation was estimated based on reported average half-life of *E. coli* mRNAs of around 10 min (10).

Recombination by Int and Int+RDF are described by a system of 35 ordinary differential equations (ODEs) for intermediate complexes between Int, Int-RDF and DNA (PB or LR; Figure S8) (8). The equations also include the production of Int and RDF proteins and their dilution as follows:

$$\begin{aligned}\frac{d[\mathrm{LR}]}{dt} &= k_{+b2}\cdot[\mathrm{LR\text{-}int2}] - k_{-b2}\cdot[\mathrm{LR}]\cdot[\mathrm{int2}] + k_{-b3}\cdot([\mathrm{LR\text{-}int2\text{-}rdf}]+[\mathrm{LR\text{-}int2\text{-}rdf2}]) - k_{+}\cdot[\mathrm{LR}]\cdot \\ &([\mathrm{int2\text{-}rdf}]+[\mathrm{int2\text{-}rdf2}]) + k_{dil}\cdot([\mathrm{LR\text{-}int2}]+[\mathrm{LR\text{-}int4}]+[\mathrm{LR\text{-}int2\text{-}rdf}]+[\mathrm{LR\text{-}int2\text{-}rdf2}]+[\mathrm{LR\text{-}int4\text{-}rdf}]+ \\ &[\mathrm{LR\text{-}int4\text{-}rdf2}]+[\mathrm{LR\text{-}int4\text{-}rdf3}]+[\mathrm{LR\text{-}int4\text{-}rdf4}]+[\mathrm{LR\text{-}int\text{-}rdf_s}]+[\mathrm{LR\text{-}int_{s1}}]+[\mathrm{LR\text{-}int_{s2}}])\end{aligned} \tag{1}$$

$$\frac{d[\mathrm{int}]}{dt} = k_{\mathrm{int}}\cdot ara(t) + k_{-ii}\cdot(2\cdot[\mathrm{int2}]+[\mathrm{int2\text{-}rdf}]) + k_{-ir}\cdot[\mathrm{int\text{-}rdf}] - k_{+}\cdot(2\cdot[\mathrm{int}]^2+[\mathrm{int}]\cdot[\mathrm{int\text{-}rdf}]+[\mathrm{int}]\cdot[\mathrm{rdf}]) - k_{dil}\cdot[\mathrm{int}] \tag{2}$$

$$\frac{d[\mathrm{int\text{-}rdf}]}{dt} = k_{+}\cdot([\mathrm{int}]\cdot[\mathrm{rdf}]-[\mathrm{int}]\cdot[\mathrm{int\text{-}rdf}]-2\cdot[\mathrm{int\text{-}rdf}]^2) - k_{-ir}\cdot[\mathrm{int\text{-}rdf}] + k_{-ii}\cdot([\mathrm{int2\text{-}rdf}]+2\cdot[\mathrm{int2\text{-}rdf2}]) - k_{dil}\cdot[\mathrm{int\text{-}rdf}] \tag{3}$$

$$\begin{aligned}\frac{d[\mathrm{int2}]}{dt} &= k_{+}\cdot[\mathrm{int}]^2 - k_{-ii}\cdot[\mathrm{int2}] - k_{+}\cdot[\mathrm{int2}]\cdot([\mathrm{rdf}]+[\mathrm{PB}]+[\mathrm{PB\text{-}int2}]) - k_{-b2}\cdot[\mathrm{int2}]\cdot([\mathrm{LR}]+[\mathrm{LR\text{-}int2}]) - \\ &k_{+s0}\cdot[\mathrm{int2}]\cdot([\mathrm{LR\text{-}int2\text{-}rdf}]+[\mathrm{LR\text{-}int2\text{-}rdf2}]+[\mathrm{PB\text{-}int2\text{-}rdf}]+[\mathrm{PB\text{-}int2\text{-}rdf2}]) + k_{-ir}\cdot[\mathrm{int2\text{-}rdf}] + \\ &k_{-b1}\cdot([\mathrm{PB\text{-}int2}]+[\mathrm{PB\text{-}int4}]) + k_{-s01}\cdot([\mathrm{LR\text{-}int4\text{-}rdf}]+[\mathrm{LR\text{-}int4\text{-}rdf2}]) + k_{-s02}\cdot([\mathrm{PB\text{-}int4\text{-}rdf}]+[\mathrm{PB\text{-}int4\text{-}rdf2}]) + \\ &k_{+b2}\cdot([\mathrm{LR\text{-}int2}]+[\mathrm{LR\text{-}int4}]) - k_{+i}\cdot[\mathrm{int2}]\cdot([\mathrm{PB\text{-}int4}]+[\mathrm{PB\text{-}int4\text{-}rdf}]+[\mathrm{PB\text{-}int4\text{-}rdf2}]+[\mathrm{PB\text{-}int4\text{-}rdf3}]+ \\ &[\mathrm{PB\text{-}int4\text{-}rdf4}]) + k_{-i}\cdot([\mathrm{PB\text{-}int6_i}]+[\mathrm{PB\text{-}int6\text{-}rdf_i}]+[\mathrm{PB\text{-}int6\text{-}rdf2_i}]+[\mathrm{PB\text{-}int6\text{-}rdf3_i}]+[\mathrm{PB\text{-}int6\text{-}rdf4_i}]) - k_{dil}\cdot[\mathrm{int2}]\end{aligned} \tag{4}$$

$$\begin{aligned}\frac{d[\mathrm{int2\text{-}rdf}]}{dt} &= k_{+}\cdot([\mathrm{int2}]\cdot[\mathrm{rdf}]+[\mathrm{int}]\cdot[\mathrm{int\text{-}rdf}]) - (k_{-ir}+k_{-ii})\cdot[\mathrm{int2\text{-}rdf}] - k_{+}\cdot[\mathrm{int2\text{-}rdf}]\cdot[\mathrm{rdf}] + k_{-ir}\cdot[\mathrm{int2\text{-}rdf2}] - \\ &k_{+}\cdot[\mathrm{int2\text{-}rdf}]\cdot([\mathrm{PB}]+[\mathrm{LR}]) - k_{+s0}\cdot[\mathrm{int2\text{-}rdf}]\cdot([\mathrm{LR\text{-}int2}]+[\mathrm{LR\text{-}int2\text{-}rdf}]+[\mathrm{LR\text{-}int2\text{-}rdf2}]+[\mathrm{PB\text{-}int2}]+ \\ &[\mathrm{PB\text{-}int2\text{-}rdf}]+[\mathrm{PB\text{-}int2\text{-}rdf2}]) + k_{+b4}\cdot[\mathrm{PB\text{-}int2\text{-}rdf}] + k_{-s01}\cdot([\mathrm{LR\text{-}int4\text{-}rdf}]+[\mathrm{LR\text{-}int4\text{-}rdf2}]+ \\ &[\mathrm{LR\text{-}int4\text{-}rdf3}]) + k_{-s02}\cdot([\mathrm{PB\text{-}int4\text{-}rdf}]+[\mathrm{PB\text{-}int4\text{-}rdf2}]+[\mathrm{PB\text{-}int4\text{-}rdf3}]) + k_{-b3}\cdot[\mathrm{LR\text{-}int2\text{-}rdf}] - k_{dil}\cdot[\mathrm{int2\text{-}rdf}]\end{aligned} \tag{5}$$

$$\frac{d[\mathrm{int2-rdf2}]}{dt} = k_{+}\cdot([\mathrm{int2-rdf}]\cdot[\mathrm{rdf}]+[\mathrm{int-rdf}]^{2}) - (k_{-ir}+k_{-ii})\cdot[\mathrm{int2-rdf2}] - k_{+}\cdot[\mathrm{int2-rdf2}]\cdot([\mathrm{PB}]+[\mathrm{LR}]+ [\mathrm{PB-int2-rdf2}]+[\mathrm{LR-int2-rdf2}]) - k_{+s01}\cdot[\mathrm{int2-rdf2}]\cdot([\mathrm{LR-int2}]+[\mathrm{LR-int2-rdf}]+([\mathrm{PB-int2}]+ [\mathrm{PB-int2-rdf}]) + k_{+bi4}\cdot([\mathrm{PB-int2-rdf2}]+[\mathrm{PB-int4-rdf4}]) + k_{-s01}\cdot([\mathrm{LR-int4-rdf2}]+[\mathrm{LR-int4-rdf3}]) + k_{-s02}\cdot([\mathrm{PB-int4-rdf2}]+[\mathrm{PB-int4-rdf3}]) + k_{-bi3}\cdot([\mathrm{LR-int2-rdf2}]+[\mathrm{LR-int4-rdf4}]) - k_{dil}\cdot[\mathrm{int2-rdf2}] \quad (6)$$

$$\frac{d[\mathrm{rdf}]}{dt} = k_{rdf\_tsl}\cdot[\mathrm{rdf_m}] + k_{-ir}\cdot([\mathrm{int-rdf}]+[\mathrm{int2-rdf}]+[\mathrm{int2-rdf2}]+[\mathrm{BP-int2-rdf}]+[\mathrm{LR-int2-rdf}]+[\mathrm{PB-int2-rdf2}]+ [\mathrm{LR-int2-rdf2}]) - k_{+}\cdot[\mathrm{rdf}]\cdot([\mathrm{int}]+[\mathrm{int2}]+[\mathrm{int2-rdf}]+[\mathrm{PB-int2}]+[\mathrm{LR-int2}]+[\mathrm{PB-int2-rdf}]+[\mathrm{LR-int2-rdf}]) - k_{dil}\cdot[\mathrm{rdf}] \quad (7)$$

$$\frac{d[\mathrm{PB-int2}]}{dt} = k_{+}\cdot[\mathrm{int2}]\cdot[\mathrm{PB}] - k_{-b1}\cdot[\mathrm{PB-int2}] - k_{+}\cdot[\mathrm{PB-int2}]\cdot[\mathrm{int2}] - k_{+s0}\cdot[\mathrm{PB-int2}]\cdot ([\mathrm{int2-rdf}]+[\mathrm{int2-rdf2}]) + k_{-b1}\cdot[\mathrm{PB-int4}] + k_{-s02}\cdot([\mathrm{PB-int4-rdf}]+[\mathrm{PB-int4-rdf2}]) - k_{+}\cdot[\mathrm{rdf}]\cdot[\mathrm{PB-int2}] - k_{-ir}\cdot[\mathrm{PB-int2-rdf}] - k_{dil}\cdot[\mathrm{PB-int2}] \quad (8)$$

$$\frac{d[\mathrm{LR-int2}]}{dt} = k_{-b2}\cdot[\mathrm{int2}]\cdot([\mathrm{LR}]-[\mathrm{LR-int2}]) - k_{+b2}\cdot([\mathrm{LR-int2}]-[\mathrm{LR-int4}]) - k_{+s0}\cdot[\mathrm{LR-int2}]\cdot([\mathrm{int2-rdf}] + [\mathrm{int2-rdf2}]) + k_{-s01}\cdot([\mathrm{LR-int4-rdf}]+[\mathrm{LR-int4-rdf2}]) - k_{+}\cdot[\mathrm{rdf}]\cdot[\mathrm{LR-int2}] + k_{+ir}\cdot[\mathrm{LR-int2-rdf}] - k_{dil}\cdot[\mathrm{LR-int2}] \quad (9)$$

$$\frac{d[\mathrm{PB-int4}]}{dt} = k_{+}\cdot[\mathrm{int2}]\cdot[\mathrm{PB-int2}] - k_{-b1}\cdot[\mathrm{PB-int4}] - k_{+s}\cdot[\mathrm{PB-int4}] + k_{-s1}\cdot[\mathrm{PB-int_s}] - k_{+i}\cdot[\mathrm{int2}]\cdot[\mathrm{PB-int4}] + k_{-i}\cdot[\mathrm{PB-int6_i}] - k_{dil}\cdot[\mathrm{PB-int4}] \quad (10)$$

$$\frac{d[\mathrm{LR-int4}]}{dt} = k_{-b2}\cdot[\mathrm{int2}]\cdot[\mathrm{LR-int2}] - k_{+b2}\cdot[\mathrm{LR-int4}] - k_{-s2}\cdot[\mathrm{LR-int4}] + k_{+s2}\cdot[\mathrm{LR-int_{s2}}] - k_{dil}\cdot[\mathrm{LR-int4}] \quad (11)$$

$$\frac{d[\mathrm{LR-int_{s2}}]}{dt} = k_{-s2}\cdot[\mathrm{LR-int4}] - k_{+s2}\cdot[\mathrm{LR-int_{s2}}] + k_{+\mathrm{mod}}\cdot[\mathrm{LR-int_{s1}}] - k_{-\mathrm{mod}}\cdot[\mathrm{LR-int_{s2}}] - k_{dil}\cdot[\mathrm{LR-int2_{s2}}] \quad (12)$$

$$\frac{d[\mathrm{PB-int_s}]}{dt} = k_{+s}\cdot[\mathrm{PB-int4}] - k_{-s1}\cdot[\mathrm{PB-int_s}] - k_{+r}\cdot[\mathrm{PB-int_s}] + k_{-r}\cdot[\mathrm{LR-int_{s1}}] - k_{dil}\cdot[\mathrm{PB-int_s}] \quad (13)$$

$$\frac{d[\mathrm{LR-int_{s1}}]}{dt} = k_{+r}\cdot[\mathrm{PB-int_s}] - k_{-r}\cdot[\mathrm{LR-int_{s1}}] - k_{+\mathrm{mod}}\cdot[\mathrm{LR-int_{s1}}] + k_{-\mathrm{mod}}\cdot[\mathrm{LR-int_{s2}}] - k_{dil}\cdot[\mathrm{LR-int2_{s1}}] \quad (14)$$

$$\frac{d[\mathrm{PB-int2-rdf}]}{dt} = k_{+}\cdot[\mathrm{int2-rdf}]\cdot[\mathrm{PB}] - k_{+b4}\cdot[\mathrm{PB-int2-rdf}] - k_{+s0}\cdot[\mathrm{PB-int2-rdf}]\cdot([\mathrm{int2}]+ [\mathrm{int2-rdf}]+[\mathrm{int2-rdf2}]) + k_{-s02}\cdot([\mathrm{PB-int4-rdf}]+[\mathrm{PB-int4-rdf2}]+[\mathrm{PB-int4-rdf3}]) + k_{+}\cdot[\mathrm{rdf}]\cdot([\mathrm{PB-int2}]-[\mathrm{PB-int2-rdf}]) - k_{-ir}\cdot([\mathrm{PB-int2-rdf}]-[\mathrm{PB-int2-rdf2}]) - k_{dil}\cdot[\mathrm{PB-int2-rdf}] \quad (15)$$

$$\frac{d[\mathrm{LR-int2-rdf}]}{dt} = k_{+}\cdot[\mathrm{int2-rdf}]\cdot[\mathrm{LR}] - k_{-b3}\cdot[\mathrm{LR-int2-rdf}] - k_{+s0}\cdot[\mathrm{LR-int2-rdf}]\cdot([\mathrm{int2}]+ [\mathrm{int2-rdf}]+[\mathrm{int2-rdf2}]) + k_{-s01}\cdot([\mathrm{LR-int4-rdf}]+[\mathrm{LR-int4-rdf2}]+[\mathrm{LR-int4-rdf3}]) + k_{+}\cdot[\mathrm{rdf}]\cdot([\mathrm{LR-int2}]-[\mathrm{LR-int2-rdf}]) - k_{-ir}\cdot([\mathrm{LR-int2-rdf}]-[\mathrm{LR-int2-rdf2}]) - k_{dil}\cdot[\mathrm{LR-int2-rdf}] \quad (16)$$

$$\frac{d[\mathrm{PB-int2-rdf2}]}{dt} = k_{+}\cdot[\mathrm{int2-rdf2}]\cdot[\mathrm{PB}] - k_{+b4}\cdot[\mathrm{PB-int2-rdf2}] + k_{+}\cdot[\mathrm{PB-int2-rdf}]\cdot[\mathrm{rdf}] - k_{+ir}\cdot[\mathrm{PB-int2-rdf2}] - k_{+}\cdot[\mathrm{PB-int2-rdf2}]\cdot[\mathrm{int2-rdf2}] + k_{+b4}\cdot[\mathrm{PB-int4-rdf4}] - k_{+s0}\cdot[\mathrm{PB-int2-rdf2}]\cdot ([\mathrm{int2}]+[\mathrm{int2-rdf}]) + k_{-s02}\cdot([\mathrm{PB-int4-rdf2}]+[\mathrm{PB-int4-rdf3}]) - k_{dil}\cdot[\mathrm{PB-int2-rdf2}] \quad (17)$$

$$\frac{d[\mathrm{LR-int2-rdf2}]}{dt} = k_{+}\cdot[\mathrm{int2-rdf2}]\cdot[\mathrm{LR}] - k_{-b3}\cdot[\mathrm{LR-int2-rdf2}] + k_{+}\cdot[\mathrm{LR-int2-rdf}]\cdot[\mathrm{rdf}] - k_{+ir}\cdot[\mathrm{LR-int2-rdf2}] - k_{+}\cdot[\mathrm{LR-int2-rdf2}]\cdot[\mathrm{int2-rdf2}] + k_{-b3}\cdot[\mathrm{LR-int4-rdf4}] - k_{+s0}\cdot[\mathrm{LR-int2-rdf2}]\cdot ([\mathrm{int2}]+[\mathrm{int2-rdf}]) + k_{-s01}\cdot([\mathrm{LR-int4-rdf2}]+[\mathrm{LR-int4-rdf3}]) - k_{dil}\cdot[\mathrm{LR-int2-rdf2}] \quad (18)$$

$$\frac{d[\text{PB-int4-rdf4}]}{dt} = k_{+} \cdot [\text{int2-rdf2}] \cdot [\text{PB-int2-rdf2}] - k_{-b4} \cdot [\text{PB-int4-rdf4}] - k_{-s4} \cdot [\text{PB-int4-rdf4}] + k_{+s4} \cdot [\text{PB-int-rdf}_{\text{s2}}] - k_{+i} \cdot [\text{int2}] \cdot [\text{PB-int4-rdf4}] + k_{-i} \cdot [\text{PB-int6-rdf4}_{\text{i}}] - k_{dil} \cdot [\text{PB-int4-rdf4}] \quad (19)$$

$$\frac{d[\text{LR-int4-rdf4}]}{dt} = k_{+} \cdot [\text{int2-rdf2}] \cdot [\text{LR-int2-rdf2}] - k_{-b3} \cdot [\text{LR-int4-rdf4}] - k_{+s} \cdot [\text{LR-int4-rdf4}] + k_{-s3} \cdot [\text{LR-int-rdf}_{\text{s}}] - k_{dil} \cdot [\text{LR-int4-rdf4}] \quad (20)$$

$$\frac{d[\text{PB-int-rdf}_{\text{s2}}]}{dt} = k_{+\text{mod}} \cdot [\text{PB-int-rdf}_{\text{s1}}] - k_{-\text{mod}\,r} \cdot [\text{PB-int-rdf}_{\text{s2}}] - k_{+s4} \cdot [\text{PB-int-rdf}_{\text{s2}}] + k_{-s4} \cdot [\text{PB-int4-rdf4}] - k_{dil} \cdot [\text{PB-int-rdf}_{\text{s2}}] \quad (21)$$

$$\frac{d[\text{PB-int-rdf}_{\text{s1}}]}{dt} = k_{+r} \cdot [\text{LR-int-rdf}_{\text{s}}] - k_{-r} \cdot [\text{PB-int-rdf}_{\text{s1}}] + k_{-\text{mod}\,r} \cdot [\text{PB-int-rdf}_{\text{s2}}] - k_{+\text{mod}} \cdot [\text{PB-int-rdf}_{\text{s1}}] - k_{dil} \cdot [\text{PB-int2-rdf}_{\text{s1}}] \quad (22)$$

$$\frac{d[\text{LR-int-rdf}_{\text{s}}]}{dt} = k_{+s} \cdot [\text{LR-int4-rdf4}] - k_{-s3} \cdot [\text{LR-int-rdf}_{\text{s}}] - k_{+r} \cdot [\text{LR-int-rdf}_{\text{s}}] + k_{-r} \cdot [\text{PB-int-rdf}_{\text{s1}}] - k_{dil} \cdot [\text{LR-int-rdf}_{\text{s}}] \quad (23)$$

$$\frac{d[\text{PB}]}{dt} = k_{-b1} \cdot [\text{PB-int2}] + k_{+b4} \cdot ([\text{PB-int2-rdf}] + [\text{PB-int2-rdf2}]) - k_{+} \cdot [\text{PB}] \cdot ([\text{int2}] + [\text{int2-rdf}] + [\text{int2-rdf2}]) + k_{dil} \cdot ([\text{PB-int2}] + [\text{PB-int4}] + [\text{PB-int2-rdf}] + [\text{PB-int2-rdf2}] + [\text{PB-int4-rdf}] + [\text{PB-int4-rdf2}] + [\text{PB-int4-rdf3}] + [\text{PB-int4-rdf4}] + [\text{PB-int-rdf}_{\text{s1}}] + [\text{PB-int-rdf}_{\text{s2}}] + [\text{PB-int}_{\text{s}}] + [\text{PB-int6}_{\text{i}}] + [\text{PB-int6-rdf}_{\text{i}}] + [\text{PB-int6-rdf2}_{\text{i}}] + +[\text{PB-int6-rdf3}_{\text{i}}] + [\text{PB-int6-rdf4}_{\text{i}}]) \quad (24)$$

$$\frac{d[\text{PB-int4-rdf}]}{dt} = k_{+s0} \cdot ([\text{int2}] \cdot [\text{PB-int2-rdf}] + [\text{int2-rdf}] \cdot [\text{PB-int2}]) - 2k_{-s02} \cdot [\text{PB-int4-rdf}] - k_{+i} \cdot [\text{int2}] \cdot [\text{PB-int4-rdf}] + k_{-i} \cdot [\text{PB-int6-rdf}_{\text{i}}] - k_{dil} \cdot [\text{PB-int4-rdf}] \quad (25)$$

$$\frac{d[\text{PB-int4-rdf2}]}{dt} = k_{+s0} \cdot ([\text{int2}] \cdot [\text{PB-int2-rdf2}] + [\text{int2-rdf}] \cdot [\text{PB-int2-rdf}] + [\text{int2-rdf2}] \cdot [\text{PB-int2}]) - 3k_{-s02} \cdot [\text{PB-int4-rdf2}] - k_{+i} \cdot [\text{int2}] \cdot [\text{PB-int4-rdf2}] + k_{-i} \cdot [\text{PB-int6-rdf2}_{\text{i}}] - k_{dil} \cdot [\text{PB-int4-rdf2}] \quad (26)$$

$$\frac{d[\text{PB-int4-rdf3}]}{dt} = k_{+s0} \cdot ([\text{int2-rdf}] \cdot [\text{PB-int2-rdf2}] + [\text{int2-rdf2}] \cdot [\text{PB-int2-rdf}]) - 2k_{-s02} \cdot [\text{PB-int4-rdf3}] - k_{+i} \cdot [\text{int2}] \cdot [\text{PB-int4-rdf3}] + k_{-i} \cdot [\text{PB-int6-rdf3}_{\text{i}}] - k_{dil} \cdot [\text{PB-int4-rdf3}] \quad (27)$$

$$\frac{d[\text{LR-int4-rdf}]}{dt} = k_{+s0} \cdot ([\text{int2}] \cdot [\text{LR-int2-rdf}] + [\text{int2-rdf}] \cdot [\text{LR-int2}]) - 2k_{-s01} \cdot [\text{LR-int4-rdf}] - k_{dil} \cdot [\text{LR-int4-rdf}] \quad (28)$$

$$\frac{d[\text{LR-int4-rdf2}]}{dt} = k_{+s0} \cdot ([\text{int2}] \cdot [\text{LR-int2-rdf2}] + [\text{int2-rdf}] \cdot [\text{LR-int2-rdf}] + [\text{int2-rdf2}] \cdot [\text{LR-int2}]) - 3k_{-s01} \cdot [\text{LR-int4-rdf2}] - k_{dil} \cdot [\text{LR-int4-rdf2}] \quad (29)$$

$$\frac{d[\text{LR-int4-rdf3}]}{dt} = k_{+s0} \cdot ([\text{int2-rdf}] \cdot [\text{LR-int2-rdf2}] + [\text{int2-rdf2}] \cdot [\text{LR-int2-rdf}]) - 2k_{-s01} \cdot [\text{LR-int4-rdf3}] - k_{dil} \cdot [\text{LR-int4-rdf3}] \quad (30)$$

$$\frac{d[\text{PB-int6}_{\text{i}}]}{dt} = k_{+i} \cdot [\text{int2}] \cdot [\text{PB-int4}] - k_{-i} \cdot [\text{PB-int6}_{\text{i}}] - k_{dil} \cdot [\text{PB-int6}_{\text{i}}] \quad (31)$$

$$\frac{d[\text{PB-int6-rdf4}_{\text{i}}]}{dt} = k_{+i} \cdot [\text{int2}] \cdot [\text{PB-int4-rdf4}] - k_{-i} \cdot [\text{PB-int6-rdf4}_{\text{i}}] - k_{dil} \cdot [\text{PB-int6-rdf4}_{\text{i}}] \quad (32)$$

$$\frac{d[\mathrm{PB-int6-rdf_i}]}{dt} = k_{+i} \cdot [\mathrm{int2}] \cdot [\mathrm{PB-int4-rdf}] - k_{-i} \cdot [\mathrm{PB-int6-rdf_i}] - k_{dil} \cdot [\mathrm{PB-int6-rdf_i}] \quad (33)$$

$$\frac{d[\mathrm{PB-int6-rdf2_i}]}{dt} = k_{+i} \cdot [\mathrm{int2}] \cdot [\mathrm{PB-int4-rdf2}] - k_{-i} \cdot [\mathrm{PB-int6-rdf2_i}] - k_{dil} \cdot [\mathrm{PB-int6-rdf2_i}] \quad (34)$$

$$\frac{d[\mathrm{PB-int6-rdf3_i}]}{dt} = k_{+i} \cdot [\mathrm{int2}] \cdot [\mathrm{PB-int4-rdf3}] - k_{-i} \cdot [\mathrm{PB-int6-rdf3_i}] - k_{dil} \cdot [\mathrm{PB-int6-rdf3_i}] \quad (35)$$

For the genetic circuit shown in Main Text Figure 5D, the transcription of *rdf* and *tetR* genes to produce messenger RNAs (rdf_m and $tetR_m$ respectively), and the production of TetR protein by translation of $tetR_m$ are described by the following equations:

$$\frac{d[\mathrm{rdf_m}]}{dt} = k_{tscr} \cdot [\mathrm{LR_{tot}}]/(1+([\mathrm{tetR}]/\mathrm{K_{tet}})^2) - k_{rna} \cdot [\mathrm{rdf_m}] \quad (36)$$

$$\frac{d[\mathrm{tetR_m}]}{dt} = k_{tscr} \cdot [\mathrm{PB_{tot}}] - k_{rna} \cdot [\mathrm{tetR_m}] \quad (37)$$

$$\frac{d[\mathrm{tetR}]}{dt} = k_{tet\_tsl} \cdot [\mathrm{tetR_m}] - k_{dil} \cdot [\mathrm{tetR}] \quad (38)$$

The equations use the following symbols [int], [int2], [rdf], [int2-rdf], [int2-rdf2] and [tetR] are the concentrations of Int, its dimers, RDF, complexes of an Int dimer with one or two molecules of RDF and TetR protein; [rdf_m], [$tetR_m$] are the concentrations of *rdf* and *tetR* mRNA; [LR], [PB] are concentrations of free DNA; [PB_{tot}] and [LR_{tot}] are the total concentrations of all DNA-containing species containg PB and LR DNA respectively, with the total DNA concentration being estimated as 17 nM assuming that the number of plasmids just after cell division is 10 plasmid copies per cell, and the cell volume is 10^{-15} l.

[PB-int2], [LR-int2] are concentrations of DNA complexes with only one site occupied by an integrase dimer (Figure S8); [PB-int2-rdf], [PB-int2-rdf2], [LR-int2-rdf], and [LR-int2-rdf2] are concentrations of complexes containing an integrase dimer and one or two RDF molecules; [PB-int4], [LR-int4], [PB-int4-rdf4], [LR-int4-rdf4] are the concentrations of DNA complexes consisting of two integrase dimers (not synapsed) with or without 4 molecules of RDF; [PB-int_s], [LR-int_{s1}], [LR-int_{s2}], [LR-int-rdf_s], [PB-int-rdf_{s1}], [PB-int-rdf_{s2}] are the concentration of integrase synapses; [PB-int4-rdf], [PB-int4-rdf2], [PB-int4-rdf3], [LR-int4-rdf], [LR-int4-rdf2], [LR-int4-rdf3] are the concentrations of unproductive complexes of Int, RDF and DNA, forming when concentration of RDF is not sufficient for recombination; PB-$int6_i$, PB-int6-rdf_i, PB-int6-$rdf2_i$, PB-int6-$rdf3_i$, PB-int6-$rdf4_i$ are non-productive Int complexes forming at high Int concentrations. *ara(t)* is a step function (11), describing the kinetics of the arabinose pulse, in dimensionless units:

$$ara(t) = 0.5 \cdot (\tanh((t - per \cdot floor(t/per) - ara_{on})/k_t) - \tanh((t - per \cdot floor(t/per) - ara_{off})/k_t)),$$

where ara_{on} and ara_{off} determine the times of the beginning and end of each pulse of inducer, administrated with a period *per* (*per* = 24 h, except where varied as stated in the legend for Supplementary Figure S14) with a characteristic time k_t = 0.3 h of arabinose dilution due to cell division. The switch without TetR (equivalent to pSWITCH2 in DS941, with a constitutive promoter for RDF expression, Main Text Figure 5A) was modelled using the same equations as the switch with TetR (equations above), but without the inhibitory term in eq. 36. The parameters are presented in Supplementary Table S1. Most of parameters were taken from the existing literature and the rate constants of the production of Int, RDF and TetR proteins k_{int}, k_{rdf_tsl} and k_{tet_tsl} were chosen to match our data. The unit of concentration is μM, and the unit of time is an hour. The system of ODEs was solved using MATLAB, integrated with the stiff solver ode15s (MathWorks UK, Cambridge). MATLAB code of the model is available as Supplementary Data for this paper available at NAR ONLINE or can be downloaded from https://github.com/alex297/model-of-binary-counter-based-on-recombination-with-serine-integrase.

Kinetics of the counter

Initially we designed a simpler version of the counter without TetR (Main Text Figure 5A). However, this counter failed to complete the PB→LR transition due to quick accumulation of RDF after initiation of switching (Main Text Figure 5B). Therefore, we incorporated TetR expression into the counter design (Main Text Figure 5D) to delay expression of RDF during the PB→LR transition (Main Text Figure 5E). The reverse, LR→PB transition was performed by both versions of the counter (with and without TetR) equally well (Main Text Figure 5C,F).

The modelled long-term kinetics of our final counter with TetR controlled by the switch is shown in Figure S11, for different initial conditions ((A) 100% PB, (B) 100% LR, (C) and a mixture of PB and LR). Independent of the initial conditions, after several sequential PB→LR and LR→PB transitions the counter fluctuates between two stable states with: 1) low LR (30%), low RDF and high TetR, and 2) high LR (88%), high RDF and low TetR (Figure S11D). The high LR state can be attained because TetR protein produced from DNA in the PB state prevents RDF expression, stopping the LR→PB reverse reaction until TetR levels have decayed. Thus for instance, starting from 40% PB an arabinose pulse results in the transition to the LR and not the PB state (Figure S11C).

The free parameters of the switch (the rate constants of Int, RDF, TetR proteins production, k_{int}, k_{rdf_tsl}, k_{tet_tsl}) were fitted to our data on the percentage of LR DNA in both states. Next, we analysed potential ways of improving the counter efficiency by variation of RDF and TetR translation rates (k_{rdf_tsl} and k_{tetR_tsl}; Figure S10). We found a zone of high efficiency for both transitions (red area on Figure S10C). Simultaneous changes in k_{rdf_tsl} and k_{tetR_tsl} in this zone could slightly increase the efficiency. For example, our 70% LR→PB and 88% PB→LR efficiencies were reached with $k_{rdf\_tsl} = 4\ h^{-1}$, $k_{tetR\_tsl} = 0.3\ h^{-1}$ (Table S1). However, further increase of the rates up to $k_{rdf\_tsl} = 800\ h^{-1}$, $k_{tetR\_tsl} = 3.4\ h^{-1}$ resulted in 83% and 85% efficiencies of the LR→PB and PB→LR transitions respectively (not shown), which slightly improves the switch performance. Similarly, a further optimisation might be achieved by adjustment of Int levels and the duration of the arabinose pulse (Figure S10D).

Stochastic modelling of plasmid segregation during cell division.

In the deterministic model described above the counter maintains its state in absence of arabinose (no Int induction), with unchanging amounts of plasmids in the PB and LR states in each cell. However, in the experimental implementation of the counter the amounts of PB and LR plasmids in individual cells drift due to random segregation of plasmid copies after recombination has stopped (Main Text, Figure 7A). Whenever this results in a cell with only PB or only LR plasmids, these cells can only produce identical progeny, containing either all PB or all LR plasmids. Our simulations below demonstrate that this property results eventually in all cells containing either plasmid DNA only in the PB state, or plasmid DNA only in the LR state (Main Text Figure 7B; Supplementary Figure S13 B,D,F).

We first carried out a simulation starting from equal initial amounts of PB and LR plasmids in each cell (10 PB and 10 LR per cell). After 70 generations, half of the cells had only PB DNA, the other half had only LR (Supplementary Figure S13 C,D). We then carried out simulations with different initial proportions of DNA in the PB and LR states (Main Text Figure 7B; Supplementary Figure S13 A,B,E,F) After 70 generations, the number of cells containing DNA in the PB or LR states accurately reflects the initial proportion of PB and LR plasmids in all cells of the starting population (Supplementary Figure S13); as expected as there is no selective pressure during random segregation. Therefore, increasing the number of cell divisions after the arabinose pulses reduces the number of cells with mixtures of DNA in PB and LR states, and allows accurate measurement of the efficiency of the switch (Main Text Figure 8). The fluorescence of cells in the segregating population was modelled using the rate of production of GFP from plasmid copies in the LR state, and dilution

of GFP by cell division. The number of cells with fluorescence levels in different bins was counted after 10, 30 and 70 generations and is shown in Main Text Figure 7B.

The model predicts that the number of cell divisions required for completed segregation increases with the copy number of the plasmid. We next used the model to estimate more precisely the number of plasmids in our experimental conditions. Based on our observation that 70 cycles of DNA replication and cell division is enough for complete segregation (Main Text Figure 7A), the model gave an estimate of 20 plasmids per daughter cell at cell division (Main Text, Figure 7B). This is consistent with the measured copy number of plasmid pSC101, which is reported as approximately 6 copies per chromosome (12,13), equivalent to ~20 copies per cell at cell division when fast growing *E. coli* cells (doubling time of ~20 minutes) have ~3-4 chromosome equivalents per cell.

The MATLAB code of the model simulating plasmid segregation in a cell population is provided as Supplementary Data for this paper available at NAR ONLINE or can be downloaded from https://github.com/alex297/model-of-binary-counter-based-on-recombination-with-serine-integrase.

Supplementary Table S1. Parameter values of the model

parameter	k_{+}	k_{-b1}	k_{-b2}	k_{-b3}	k_{+b4}
value	3600 μM^{-1} h^{-1}	72 h^{-1}	600 μM^{-1} h^{-1}	90 h^{-1}	180 h^{-1}
ref.	(8)	(8)	(8)	(8)	(8)
parameter	k_{-ii}	k_{-ir}	k_{+i}	k_{-i}	k_{+s}
value	1080 h^{-1}	180 h^{-1}	180 μM^{-1} h^{-1}	3.6 h^{-1}	48 h^{-1}
ref.	(8)	(8)	(8)	(8)	(8)
parameter	k_{+s2}	k_{+s4}	k_{-s1}	k_{-s2}	k_{-s3}
value	0.00072 h^{-1}	0.0039 h^{-1}	4.8 h^{-1}	0.006 h^{-1}	4.8 h^{-1}
ref	(8)	(8)	(8)	(8)	(8)
parameter	k_{-s4}	k_{+r}	k_{-r}	k_{+mod}	k_{-mod}
value	0.3 h^{-1}	60 h^{-1}	60 h^{-1}	60 h^{-1}	17.6 h^{-1}
ref	(8)	(8)	(8)	(8)	(8)
parameter	k_{-modr}	k_{tscr}	K_{tet}	k_{rna}	k_{dil}
value	31.6 h^{-1}	120 h^{-1}	0.01 μM	4 h^{-1}	2 h^{-1}
ref	(8)	(14)	(15)	(10)	*
parameter	k_{int}	k_{rdf_tsl}	k_{tet_tsl}	k_t	ara_{on}-ara_{off}
value	3 μM/h**	0.3 $h^{-1,**}$	4 $h^{-1,**}$	0.3 h*	0.2 h**

* estimated based on 20 min doubling time; ** fitted to our data on the counter kinetics

Plasmid Sequences

Sequences of all the plasmids used in this study are provided in genbank annotated format in a single zip file available as Supplementary Data for this paper at NAR ONLINE.

Supplementary Table S2. List of plasmid sequences.

**pSWITCH plasmids**	**Integrase and RDF expression plasmids**
pSWITCH0-PB and -LR	pBAD-INT
pSWITCH0-HC-PB and -LR	pBAD-INT-GTG
pSWITCH0-LC-PB and -LR	pBAD-INT-56
pSWITCH1-PB and -LR	pBAD-INT-106
pSWITCH2-PB and -LR	pBAD-INT-126
pSWITCH2*-PB and -LR	pBAD[INT+RDF]
pSWITCH3-PB and -LR	pTET[INT+RDF]
pSWITCH3*-PB and -LR	pTET[INT+RDF]FUS
	pRDF-HC
	pRDF-LC

References

1. Salis, H.M. (2011) The ribosome binding site calculator. Methods Enzymol, 498, 19-42.
2. Borja, G.M., Meza Mora, E., Barron, B., Gosset, G., Ramirez, O.T. and Lara, A.R. (2012) Engineering *Escherichia coli* to increase plasmid DNA production in high cell-density cultivations in batch mode. Microb Cell Fact, 11, 132.
3. Goncalves, G.A., Prazeres, D.M., Monteiro, G.A. and Prather, K.L. (2013) *De novo* creation of MG1655-derived *E. coli* strains specifically designed for plasmid DNA production. Appl Microbiol Biotechnol, 97, 611-620.
4. Colloms, S.D., Bath, J. and Sherratt, D.J. (1997) Topological selectivity in Xer site-specific recombination. Cell, 88, 855-864.
5. Colloms, S.D., McCulloch, R., Grant, K., Neilson, L. and Sherratt, D.J. (1996) Xer-mediated site-specific recombination *in vitro*. EMBO J, 15, 1172-1181.
6. McCulloch, R., Coggins, L.W., Colloms, S.D. and Sherratt, D.J. (1994) Xer-mediated site-specific recombination at *cer* generates Holliday junctions in vivo. EMBO J, 13, 1844-1855.
7. Summers, D.K. and Sherratt, D.J. (1988) Resolution of ColE1 dimers requires a DNA sequence implicated in the three-dimensional organization of the *cer* site. EMBO J, 7, 851-858.
8. Pokhilko, A., Zhao, J., Ebenhoh, O., Smith, M.C., Stark, W.M. and Colloms, S.D. (2016) The mechanism of ϕC31 integrase directionality: experimental analysis and computational modelling. Nucleic Acids Res, 44, 7360-7372.
9. Westbrook, A., Tang, X., Marshall, R., Maxwell, C.S., Chappell, J., Agrawal, D.K., Dunlop, M.J., Noireaux, V., Beisel, C.L., Lucks, J. *et al.* (2019) Distinct timescales of RNA regulators enable the construction of a genetic pulse generator. Biotechnol Bioeng.
10. Janga, S.C. and Babu, M.M. (2009) Transcript stability in the protein interaction network of *Escherichia coli*. Mol Biosyst, 5, 154-162.
11. Pokhilko, A., Fernandez, A.P., Edwards, K.D., Southern, M.M., Halliday, K.J. and Millar, A.J. (2012) The clock gene circuit in *Arabidopsis* includes a repressilator with additional feedback loops. Mol Syst Biol, 8, 574.
12. Cabello, F., Timmis, K. and Cohen, S.N. (1976) Replication control in a composite plasmid constructed by *in vitro* linkage of two distinct replicons. Nature, 259, 285-290.
13. Hasunuma, K. and Sekiguchi, M. (1977) Replication of plasmid pSC101 in *Escherichia coli* K12: requirement for *dnaA* function. Mol Gen Genet, 154, 225-230.
14. Hooshangi, S., Thiberge, S. and Weiss, R. (2005) Ultrasensitivity and noise propagation in a synthetic transcriptional cascade. Proc Natl Acad Sci U S A, 102, 3581-3586.
15. Biliouris, K., Daoutidis, P. and Kaznessis, Y.N. (2011) Stochastic simulations of the tetracycline operon. BMC Syst Biol, 5, 9.
