# Supplementary material for: A single-input binary counting module based on serine integrase site-specific recombination
Source: Nucleic Acids Res. 2019 Apr 8;47(9):4896–909. doi: 10.1093/nar/gkz245 (PMC6511857; doi:10.1093/nar/gkz245)
Supplement: Supplementary Data [file gkz245_supplemental_files.zip › Zhao_et_al_2019_matlab_code.zip!/read_me.doc]

To simulate latch kinetics - run **tetR_110219.m**; figure(1) draws kinetics, figure(2) draws phase diagram; requires ODE file **Model_tetR_110219.m**

**Fig. 5**: run tetR_110219.m with t=[0 2], k_t_=0.03 (default k_t_=0.3), ara_on_=0.5, ara_off_=0.7. For Fig. S5B,C: K_tet_=10, for Fig. 5E,F: K_tet_=0.01. Initial conditions: Fig. 5B - y0=[0*Dtot 0 0 0 0 0 0 0 0 0 0 0 0 0 0 0 0 0 0 0 0 0 0 1*Dtot 0 0 0 0 0 0 0 0 0 0 0 0 0 0], Fig. 5E - y0=[0*Dtot 0 0 0 0 0 0 0 0 0 0 0 0 0 0 0 0 0 0 0 0 0 0 1*Dtot 0 0 0 0 0 0 0 0 0 0 0 0 0.4 0.06], Fig. 5C,F - y0=[Dtot 0 0 0 0 0 0 0 0 0 0 0 0 0 0 0 0 0 0 0 0 0 1 0*Dtot 0 0 0 0 0 0 0 0 0.5 0 0 0 0 0].

**Fig. S10**: run **tetR_3D_110219.m** for Fig. S10A-C; run **tetR_3D_110219_int.m** for Fig. S10D. Requires many hours of run, for faster runs reduce t.

**Fig. S11**: run **tetR_110219.m** with t=[0 24*5], ara_on_=2.5, ara_off_=2.7. Initial conditions: Fig. S11A - y0=[0,0,0,0,0,0,0,0,0,0,0,0,0,0,0,0,0,0,0,0,0,0,0.01,Dtot,0,0,0,0,0,0,0,0,0.005,0,0,0,0.4,0.05], Fig. S11B - y0=[1*Dtot,0,0,0,0,0,0,0,0,0,0,0,0,0,0,0,0,0,0,0,0,0,0.5,0*Dtot,0,0,0,0,0,0,0,0,0.25,0,0,0,0.06,0.01], Fig. S11C - y0=[0.6*Dtot,0,0,0,0,0,0,0,0,0,0,0,0,0,0,0,0,0,0,0,0,0,0.5,0.4*Dtot,0,0,0,0,0,0,0,0,0.25,0,0,0,0.06,0.01], Fig. S11D - figure(2) under initial conditions of Fig. S11A,B,C.

For simulations of plasmid segregation:

**Fig. 6B**: run **pl_segr_110219.m**

**Fig. S13**: run **pl_segr_110219_hist.m** with LR=3/20 (Fig. S14A,B); LR=10/20 (Fig. S14C,D); LR=17/20 (Fig. S14C,D).

**Fig. S14**: run **tetR_110219.m** with ara_on_=0.5, ara_off_=0.7. Initial run - t=[0 24*6] to reach steady state; final run t=[0 24*4]; parameter period equal to 18, 14, 10, 6, 2 for panels A-E.
